# Unveiling the fate and potential neuroprotective role of neural stem/progenitor cells in multiple sclerosis

**DOI:** 10.3389/fneur.2024.1438404

**Published:** 2024-11-20

**Authors:** Nora Hijal, Malak Fouani, Bassel Awada

**Affiliations:** ^1^Department of Nursing, American University of Beirut Medical Center, Beirut, Lebanon; ^2^Department of Neurology, Duke University Medical Center, Durham, NC, United States; ^3^Department of Experimental Pathology, Immunology, and Microbiology, American University of Beirut, Beirut, Lebanon

**Keywords:** adult neurogenesis, multiple sclerosis, oligodendrogenesis, demyelination, stem cells, novel therapies, neural progenitor cells, immune system dysfunction

## Abstract

Chronic pathological conditions often induce persistent systemic inflammation, contributing to neuroinflammatory diseases like Multiple Sclerosis (MS). MS is known for its autoimmune-mediated damage to myelin, axonal injury, and neuronal loss which drive disability accumulation and disease progression, often manifesting as cognitive impairments. Understanding the involvement of neural stem cells (NSCs) and neural progenitor cells (NPCs) in the remediation of MS through adult neurogenesis (ANG) and gliogenesis—the generation of new neurons and glial cells, respectively is of great importance. Hence, these phenomena, respectively, termed ANG and gliogenesis, involve significant structural and functional changes in neural networks. Thus, the proper integration of these newly generated cells into existing circuits is not only key to understanding the CNS’s development but also its remodeling in adulthood and recovery from diseases such as MS. Understanding how MS influences the fate of NSCs/NPCs and their possible neuroprotective role, provides insights into potential therapeutic interventions to alleviate the impact of MS on cognitive function and disease progression. This review explores MS, its pathogenesis, clinical manifestations, and its association with ANG and gliogenesis. It highlights the impact of altered NSCs and NPCs’ fate during MS and delves into the potential benefits of its modifications. It also evaluates treatment regimens that influence the fate of NSCS/NPCs to counteract the pathology subsequently.

## 1 Introduction

Multiple Sclerosis (MS) is a chronic inflammatory condition affecting the central nervous system (CNS), characterized by inflammatory demyelination and neurodegeneration. Over time, the progressive accumulation of damage leads to irreversible disability, defining the advanced stages of the disease (1). MS is considered the primary cause of nontraumatic neurological disability (2) worldwide, with the most affected regions being Northern Europe and North America. For example in the United States alone, there are around 1 million people currently living with MS (3).

The definitive etiology of MS remains unclear, but its development is influenced by various risk factors. Epstein–Barr virus, exposure to Ultraviolet B waves, smoking, vitamin D levels, childhood obesity, aging, female sex, and genetic predisposition contribute to the causal pathway leading to MS (4–7). Low vitamin D levels, due to reduced intake, limited outdoor activity, or genetic polymorphisms, increase susceptibility to MS (8). Similarly, smoking raises the risk of MS by approximately 50%, particularly due to organic solvents and smoked tobacco, which influence the immunogenic environment in the lungs (5, 9, 10). Furthermore, genetic factors also play a role, with about one in eight patients having a family history of the disease. HLA-DRB1*15 and related loci are primary genetic risks associated with MS, with heterozygous individuals having an odds ratio of more than 3 for developing MS, and homozygotes having an odds ratio of more than 6. The exact mechanism behind this association is unknown (5, 11, 12). As for aging, it leads to a decline in biological functions, contributing to various comorbidities in the elderly. Immunosenescence, the decline in the immune system, causes chronic inflammation (“inflamm-aging”) with elevated inflammatory cytokines increasing morbidity risk (13). This phenomenon influences the clinical course of MS as patients with late-onset MS or LOMS (> 50 years) typically experience rapid development of permanent disability. Whereas those with pediatric-onset MS or POMS (<18 years) have a more progressive disease course (13). Nonetheless, MS patients including those with POMS, exhibit early immunosenescence since disease onset, characterized by shorter telomeres, thymic dysfunction, increased lymphocytes and memory T cells, reduced naïve T cells, and less functional regulatory T cells (Tregs) (14). However, in elderly MS patients, reduced cerebral plasticity and growth factor levels lead to incomplete recovery from demyelination and axonal degeneration, thus declining the chances of recovery. Increased blood brain barrier (BBB) permeability with aging also allows more inflammatory cell infiltration into the CNS, promoting astrocyte proliferation and glial scar development, hindering recovery, and reducing therapeutic efficacy (15). Additionally, decreased neurogenesis, compromised astrocyte support, and reduced synaptic density contribute to age-related impaired neurodegeneration (16). Neural progenitor cells (NPCs) from subjects with progressive MS express markers of cellular senescence *in situ* and *in vitro*, and their secretome induces the expression of senescence genes in oligodendrocyte progenitor cells (OPCs) and inhibits their differentiation (17).

As mentioned above, MS is an extremely complex disease with extensive effects on both the nervous and immune systems. This review focuses on deepening the understanding of MS, its pathogenesis, and clinical manifestations and considerations. It also emphasizes the involvement of Neural Stem Cells (NSCs) and Neural Progenitor Cells (NPCs), particularly in the recovery from MS by promoting neurological phenomena, namely neurogenesis and gliogenesis. These processes involve the formation of novel neurons and glia in the CNS, respectively. Additionally, we will discuss their normal occurrence, their alteration during MS, and the beneficial aspects these modifications might bring. Finally, we will examine how certain treatment regimens can influence these phenomena and consequently help combat MS.

## 2 Clinical presentation and diagnosis of MS

### 2.1 Clinical presentation

MS is an unpredictable disease with a course that varies between each individual. The International Advisory Committee on Clinical Trials of MS categorized the condition into four disease types or courses (18): Clinically Isolated Syndrome (CIS), Relapsing–Remitting MS (RRMS), Secondary Progressive MS (SPMS), andPrimary Progressive MS (PPMS).

The term CIS refers to the initial clinical event that strongly indicates a demyelinating CNS disease. Typically, the presenting symptoms of CIS are localized to a single area (monofocal), and they develop rapidly or sub-acutely over days or weeks (19, 20). The early symptoms commonly involve one or more of the following: weakness or reduced dexterity in one or more limbs, sensory disturbances, optic neuritis, diplopia, ataxia. As for RRMS, it is the most common MS type and is marked by relapse and remission cycles; it can further be classified by stability and activity levels based on lesion changes (20). Relapses vary by patient, often causing permanent deficits and gradual impairment accumulation (21). The majority of RRMS patients, specifically untreated ones, eventually transition into SPMS (22). Risk factors of conversion to SPMS, include, higher age at onset of RRMS, male gender, SC symptoms, and incomplete relapse (22). SPMS consists of periods of progression that may include relapses or stable disability phases. Diagnosis is typically based on clinical grounds throughout at least 6 to 12 months. Similarly to RRMS, SPMS is subdivided into active or not active, and with or without progression (20). Whereas for PPMS, it is estimated that approximately 10 to 20% of patients are affected by this disease phenotype, which is characterized by continuous progression from onset without a relapsing phase (23, 24). Patients’ experiences vary relapses or stability periods may occur. Growing evidence supports that PPMS and SPMS share underlying features within the MS spectrum (25).

The National MS Society and the European Committee for Treatment and Research in MS suggest a novel approach to classify MS as an ongoing disease process influenced by underlying mechanisms of nervous system damage, balanced by the individual’s ability to repair or compensate for this damage. For instance, MS could be considered a solitary disease with its spectrum ranging from relapsing to progressive. Therefore referring to MS as subtypes places patients in inaccurate divisions, whereas they should be considered on a continuum (5). Although the new classification method is promising, implementing this change will take considerable time. Hence, for the time being, researchers and healthcare providers continue to rely on the current system (26).

### 2.2 Diagnosis of MS

MS is diagnosed through a combination of clinical and radiological features, with the 2017 McDonald criteria being the standard for all forms of MS. Diagnosis involves identifying a syndrome typical of MS and finding objective evidence of CNS involvement, usually through Magnetic Resonance Imaging (MRI) of the brain and spinal cord (SC) that reveals demyelinating lesions. It also requires demonstrating dissemination in space, with lesions in multiple CNS areas, and dissemination in time, shown by multiple clinical demyelination events or MRI indicating both contrast-enhancing (acute) and non-contrast-enhancing (chronic) lesions. Additionally, there must be no better explanation for the symptoms (1, 27).

Importantly, MRI is the most effective imaging technique for predicting the progression from CIS to MS. Over a 20-year follow-up period, the risk of a second demyelinating event is 80% if typical lesions are present on the MRI, compared to 20% if they are absent. The likelihood of an early MS diagnosis (within 4 years) rises with the number of lesions, particularly with infratentorial lesions or more than three periventricular lesions. In general, about one-third of patients experience no further demyelinating events even after 30 years of follow-up (1).

## 3 Animal models

### 3.1 Experimental autoimmune encephalitis model

Although no standalone MS animal model exists, the experimental autoimmune encephalitis (EAE) model closely resembles MS in key aspects (2). MS and EAE share autoimmune mechanisms where autoreactive T-cells, especially Th1 and Th17, produce inflammatory cytokines like IFN-*γ* and IL-17, triggering CNS inflammation and tissue damage. They also show similar BBB disruptions, demyelination, and neurological deficits, with certain EAE models mimicking the relapsing–remitting course of MS, aiding in the study of MS mechanisms and therapies. Additionally, genetic and environmental factors play roles in both MS and EAE, with triggers like infections and vitamin D deficiency impacting susceptibility. However, EAE has limitations as it primarily affects the SC in rodents, whereas MS causes broader brain lesions. Despite these differences, EAE remains the closest animal model to human MS and has been instrumental in developing MS therapies (28, 29). Many EAE models exist, with the most common among them being the active EAE, which is initiated by immunizing genetically susceptible mice strains with elements of myelin such as myelin oligodendrocyte glycoprotein (MOG), myelin basic protein (MBP), or proteolipid protein (PLP) (2). A highly immunogenic adjuvant is also given to these animals to enhance the myelin autoimmunity. This model possesses multiple comparable aspects to MS at the immunological and neurological levels (30). In addition, the passive (or adoptive transfer) model, where myelin-specific CD4+ T-cells are transferred to non-immunized animals, allows for focused study on myelin-reactive T-cells in MS pathogenesis (30). Moreover, spontaneous EAE models have been established by incorporating T-cells whose receptors are reactive to autologous MOG. The transgenic animals ended up developing an EAE disease similar to RRMS (31). Therefore, it is important to note that the majority of the preclinical data seen in subsequent paragraphs were extrapolated from the EAE model.

### 3.2 Cuprizone-induced MS model

This model can be used to study the extensive demyelination of the hippocampus. Animals are fed a diet containing the copper-chelating agent Cuprizone, which kills oligodendrocytes (OLDCs), combined with Rapamycin injections, which ensure complete and consistent demyelination. This model is considered reversible as the withdrawal of Cuprizone and Rapamycin allows normal remyelination to occur (32). Thus, this model allows the visualization of the demyelination/remyelination process that can occur in certain MS patients.

### 3.3 Theiler’s murine model

Theiler’s murine encephalomyelitis virus-induced demyelinating disease (TMEV-IDD) is a significant model for studying MS as it induces chronic demyelination and inflammation in mice, closely mimicking human MS. This model involves an acute phase of viral replication and CNS inflammation, followed by a chronic phase of immune-mediated demyelination and axonal damage. TMEV-IDD research has provided crucial insights into blood–brain barrier disruption, immune cell roles, and neurodegeneration, aiding in the understanding of MS pathogenesis and therapeutic development (33, 34).

## 4 Cellular pathogenesis

### 4.1 Immunological pathogenesis of MS

**Figure 1:**
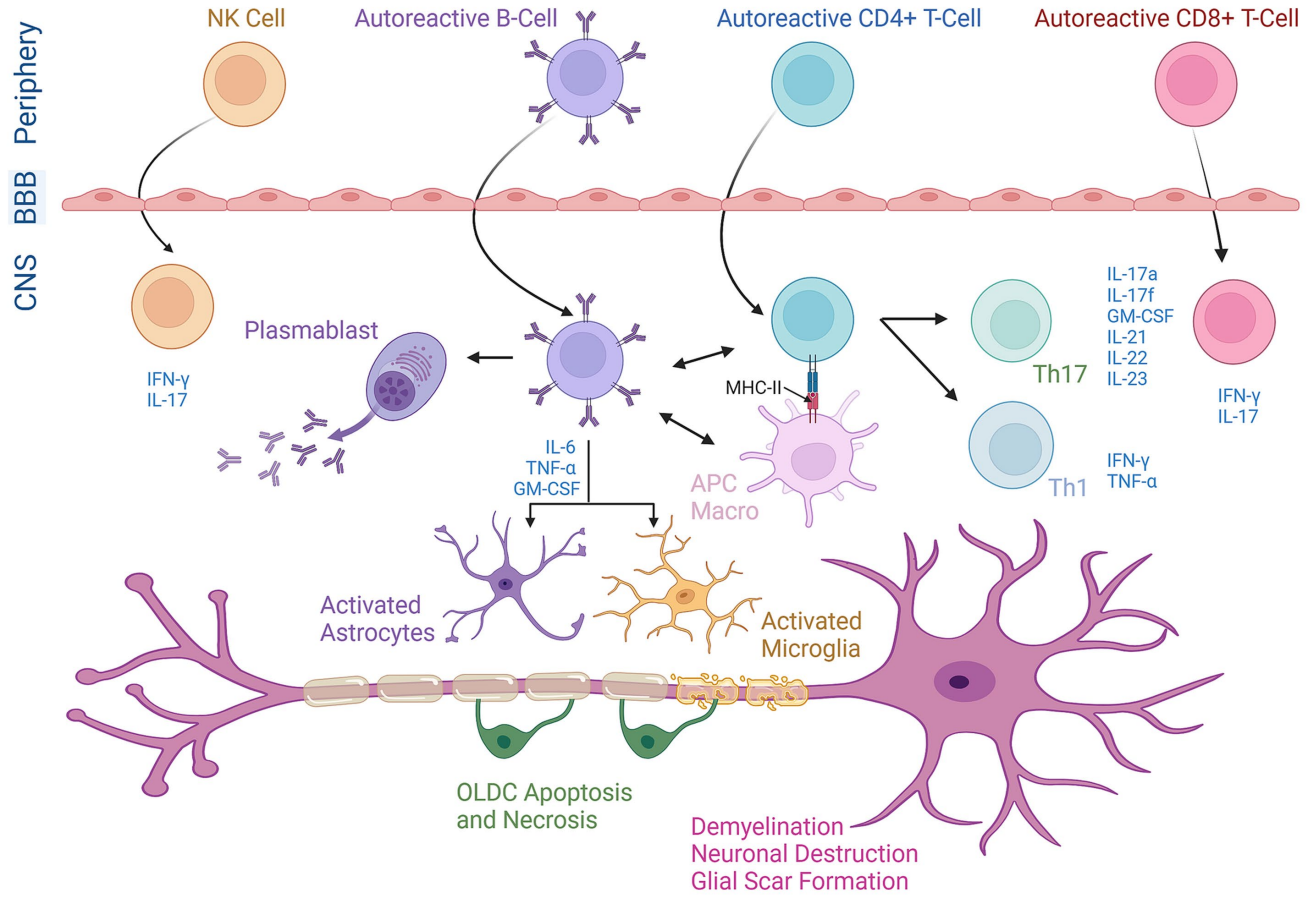
The chief role of diverse immune cells in the pathogenesis of MS. Activated T-cells (both CD4^+^ and CD8^+^ T-cells), B-cells, and Natural Killer (NK) cells originating in the periphery cross the blood–brain barrier (BBB) and enter the central nervous system (CNS). Within the CNS, these cells undergo reactivation and release cytokines as part of their effector functions. CD4+ T-cells are reactivated when MHC-II molecules on APCs or macrophages (macro) specifically present peptide anti-gens to their surface receptors and differentiate into either Th1 or Th17 phenotypes. B-cells play an active role in the progression of MS by potentially triggering the activation of Th1 cells to produce IFN-γ and TNF-*α* or Th17 cells to produce IL-17a, IL-17f, IL-21, IL-22, IL-23, and GM-CSF (granulocyte-macrophage colony-stimulating factor). Moreover, B cells co-express the pro-inflammatory cytokines GM-CSF, IL-6, and TNF-α leading to the activation and recruitment of macrophages/microglial cells and astrocytes. B-cells are further differentiated into plasma cells (plasmablasts), producing autoantibodies against myelin proteins. NK cells release IFN-γ creating a pro-inflammatory environment. This integrated neuro-inflammatory network plays an essential role in demyelination and neuronal destruction as well as oligodendrocyte (OLDC) necrosis/apoptosis in MS.

In MS, as self-tolerance breaks down, myelin-specific autoreactive T-cells are activated outside the CNS (35). These T-cells upregulate chemokines and adhesion molecules enabling them to cross the BBB and access the perivascular space (Figure 1) (36, 37). CD4^+^ T-cells are of utmost importance in the initiation and progression of MS (38). These cells move along the blood vessels, and nearby antigen-presenting cells (APCs) reinvigorate the myelin-specific T-cells (39, 40), allowing them to enter CNS tissues and target OLDCs, thus damaging the myelin sheath (41). CD4^+^ T-cell subsets with the potential to induce EAE in rodents encompass both Th1 and Th17 cells (42). Th17 cells are identified as a distinct subset of CD4^+^ T-cells that produce various inflammatory cytokines essential for MS progression, including IL-17A and IL-17F (Figure 1) (43). Whereas Th1 cells are distinguished by their production of Interferon *γ* (IFN-γ) as their hallmark cytokine. A clinical study revealed that treating MS patients with IFN-*γ* led to a significant increase in the post-treatment exacerbation rate of the disease when compared to the pretreatment rate (44). However, in EAE models, both systemic and CNS administrations of IFN-γ reduced clinical symptoms, suggesting a neuroprotective role in EAE. Moreover, the absence of IFN-*γ* or its receptor leads to atypical EAE characterized by increased Th1 and Th17 cell activity and brain inflammation, which is rarely seen in classical EAE where damage is typically spinal (45). This is suggested to be the result of IFN-*γ* causing SC inflammation while protecting the brain by regulating chemokines that limit T-cell infiltration (45). Moreover, it was shown that prevention of atypical EAE by IFN-*γ* is dependent on IFN-γ signaling not only in encephalitogenic T cells but also in glial cells (45). Low doses of IFN-*γ* protect microglia and OLDCs, while high doses can be harmful. Astrocytes typically promote disease progression through IFN-γ signaling, while OLDC responses depend on maturation and stress. These findings suggest that fluctuations in IFN-γ expression or signaling could play a significant role in the immunopathogenesis of EAE, which is variable by CNS region and cell types involved (45). Further, studies have shown the overexpression of the Th1 cytokine tumor necrosis factor-alpha (TNF-*α*) in the CNS resulted in demyelination whereas blocking TNF-α before EAE onset attenuated the disease (46). Additionally, data collected from live patients’ biopsies suggest that CD8^+^ T-cells possess a neuronal tissue-damaging role in MS (47). The effective engagement between CD8^+^ T-cells and target cells necessitates the presence of MHC-I expression controlled within neurons. MHC-I molecules are solely produced in response to potent inflammatory signals such as IFN-*γ* or TNF-*α* (48).

B-cells are also pivotal in MS progression, capable of crossing the BBB (Figure 1) and becoming long-term CNS residents (49). In MS, B-cells interact with other immune cells in the CNS to increase disease severity. For instance, they present antigens to T-cells in the CNS through MCH-II (Figure 1), with the expression of the latter being crucial for inducing EAE in mouse models (50). EAE induced by a specific isoform of MOG requires the involvement of both MOG-specific T-cells and B-cells. This reflects the necessity of B cells functioning as APCs to activate T cells, a process that defines B cell-dependent EAE (51). Functional studies show that B-cells can stimulate IFN-*γ* secretion of pathogenic Th1 cells in RRMS patients. These findings suggest that B-cells contribute to MS pathogenesis by activating T-cells, leading to enhanced CNS inflammation (52). B-cells also release various cytokines, such as IL-6, which promotes Th17 cell differentiation while inhibiting regulatory T-cells (53). MS patients show an increased frequency of memory B-cells co-expressing pro-inflammatory cytokines GM-CSF, IL-6, and TNF-*α* (Figure 1).

In addition, Natural Killer (NK) cells play a dual role in MS due to their capability to secrete both anti-inflammatory and pro-inflammatory cytokines. NK cells can induce apoptosis in target cells via Granzyme B (GrB) and perforin and release IFN-*γ* in response to unhealthy cells, potentially contributing to MS exacerbations by reactivating the immune response (54). On the other hand, immunomodulatory therapies increasing CD56bright NK cells have been linked to better treatment responses (e.g., Daclizumab and IFN-*β*), whereas a decrease in these cells correlates with relapse. As seen in Figure 1, the pro-inflammatory environment induced by NK cells might also contribute to MS exacerbations by reactivating the immune response (54).

### 4.2 Neurological pathogenesis of MS

MS is characterized by both axonal degeneration and neuronal death in active lesions from disease onset (Figure 1). Focal lesions in the brain and SC are the hallmarks of MS, evident by primary demyelination and partial preservation of axons and glial scar formation. Disease progression occurs when axonal loss exceeds CNS compensatory capability, resulting in irreversible neurological disability (55, 56). MS typically affects the WM, but recent studies have confirmed demyelination in cortical and deep matter may also occur (57).

Aside from immune cells invading the CNS, MS is emphasized by CNS residents. For instance, astrocytes produce peroxynitrite which inactivates glutamate transporters, thus limiting its uptake. Consequently, the resulting pathologically elevated levels of extracellular glutamate result in the death of OLDCs and neurons (58). Moreover, astrocytes partake in fibrillary gliosis, which accompanies lesion maturation, inhibits remyelination, and contributes to scar formation (59). On the other hand, microglia can accumulate alongside macrophages at sites of demyelination and neurodegeneration (60). These cells, along with astrocytes in MS patient specimens and EAE models, were shown to produce neurotoxic substances such as Reactive Oxygen Species (ROS), Reactive Nitrogen Species (RNS), and Nitric Oxide (NO) (61). Elevated levels of NO and its production markers (nitrate and nitrite) are elevated in the CSF, blood, and urine of MS patients. Data suggests elevated NO contributes to BBB disruption, axonal degeneration, and oligodendrocyte injury (62). Mitochondrial injury due to ROS and RNS affects WM lesions and later gray matter in the progressive MS phase, with neuronal mitochondrial dysfunction marked by mtDNA deletions (61, 63). This mitochondrial dysfunction can lead to energy imbalance, axonal degeneration, and cell death (63). Thus, these pathological mechanisms underscore MS’s complexity, highlighting the roles of microglia, astrocytes, and mitochondrial dysfunction in disease progression.

## 5 Neural stem cell/NPC fates: neurogenesis and gliogenesis

Although highly related to CNS development, the generation of new neurons and glial cells holds a great importance in adulthood. These phenomena, respectively termed neurogenesis and gliogenesis, involve significant structural and functional changes in neural networks. Thus, the proper integration of these newly generated cells into existing circuits is not only key to understanding the CNS’s development but also its remodeling in adulthood and recovery from diseases (64). ANG occurs within niches present in both the brain and the SC. In the brain, ANG only occurs in three regions: the subventricular zone (SVZ) of the lateral ventricles, the amygdala, and the dentate gyrus (DG) of the hippocampus. While in the SC, there is scarce information about this phenomenon as well as on the existence of neurogenic niches throughout its regions (65).

**Figure 2:**
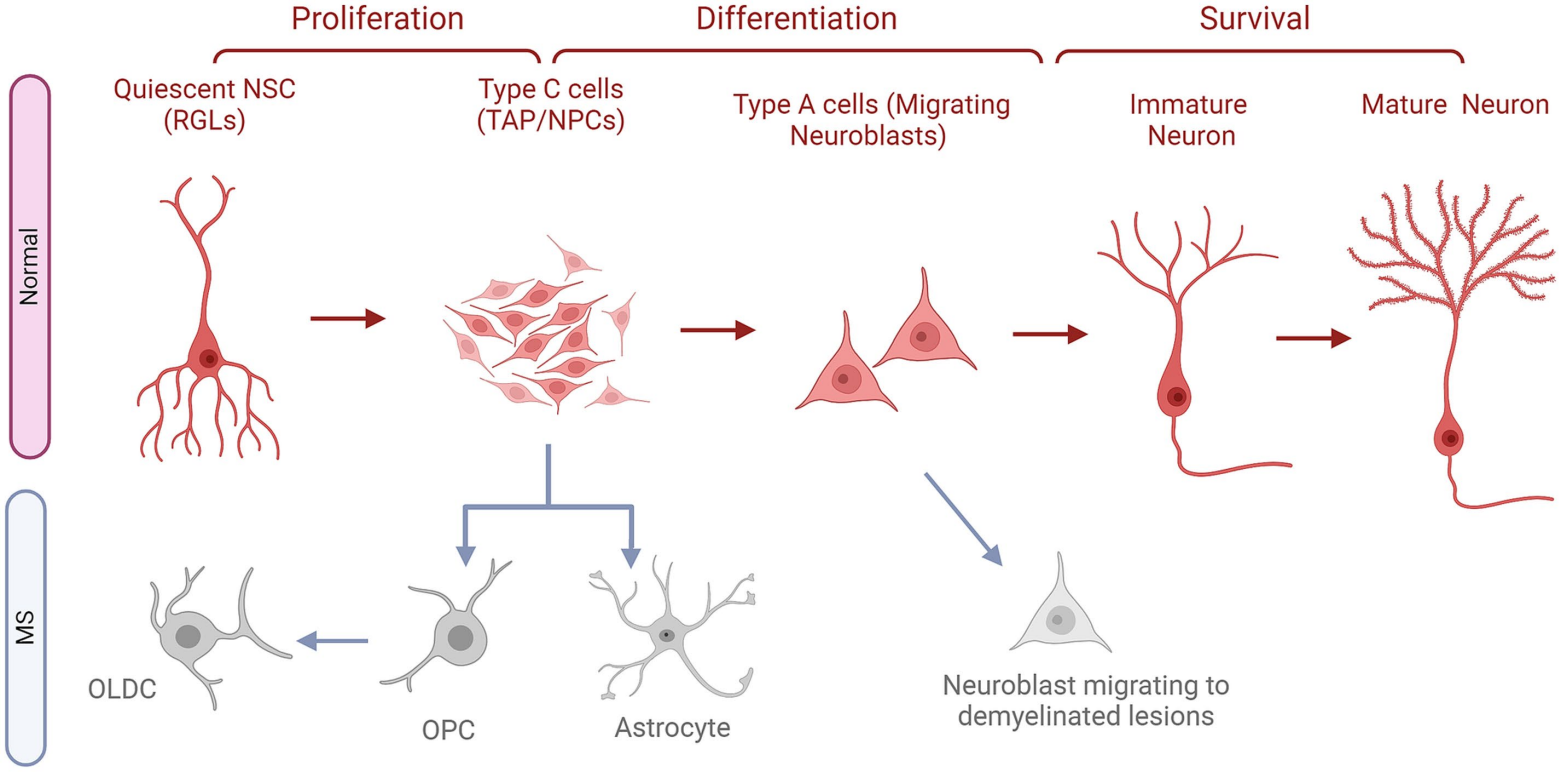
The fates of NSCs/NPCs in the SVZ in normal conditions compared to MS. Quiescent neural stem cells (NSCs or RGLs) undergo proliferation to become neural progenitor cells (NPS or C cells). These NPCs in turn, differentiate into neuroblasts, referred to as Type A cells. Following this stage, these cells migrate along the rostral migratory stream (RMS) to reach the olfactory bulb (OB). Once in the OB, they subsequently migrate radially and undergo their differentiation into first immature then mature fully developed neurons, which contribute to the OB circuitry. In MS, NPCs do not progress in their natural path instead they give rise to astrocytes and OPCs, which turn into OLDCs. Whereas, migrating neuroblasts might leave the migratory stream down the RMS and go to the demyelinated lesions to attempt repair.

SVZ-based ANG occurs when neuroblast precursors of interneurons migrate to the olfactory bulb (OB) through the rostral migratory stream or RMS (66). The NSCs persist within this niche as the continuous production of newborn neurons in this region is necessary to meet the needs of the OB (67). As seen in Figure 2, Within the adult SVZ, actively dividing NSCs also known as Radial Glia-Like cells (RGLs) generate transit amplifying cells (TAPs) or type C cells, which express the transcription factor Dlx2 (68). They subsequently give rise to type A cells (Figure 2) or migratory neuroblasts which express doublecortin (DCX) and polysialylated-neural cell adhesion molecule (PSA-NCAM) and exhibit elongated cell bodies and one or two processes (69). As neuroblasts progress through the RMS, they align in a migratory stream toward the OB, using a pathway defined by astrocytes (65, 68). Upon reaching the core of the OB, immature neurons detach from the RMS and migrate radially toward the glomeruli where they undergo differentiation into various subtypes of interneurons (65). The majority of these newly generated cells develop into GABAergic granule neurons, while a smaller proportion matures into GABAergic periglomerular neurons. Limited evidence suggests that an extremely small percentage of newly generated neurons take on the identity of glutamatergic juxtaglomerular neurons (65).

**Figure 3:**
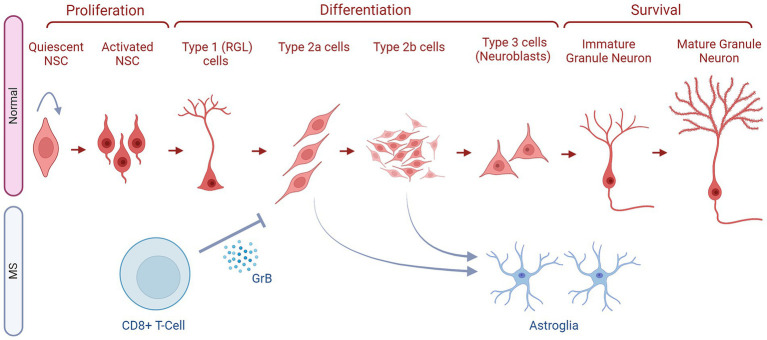
The fates of NSCs/NPCs in the hippocampus in normal conditions compared to MS. Within the SGZ niche, there is a sequence of cellular transitions. This begins with quiescent neural stem cells, which progress to activated neural stem cells (NSCs). Subsequently, they differentiate into type 1 radial glia cells (RGLs), followed by early-stage type 2a cells and type 2b cells, collectively known as neural progenitor cells (NPCs). These cells then enter a differentiation phase, leading to the generation of late-stage type 3 neuroblasts and, subsequently, immature granule neurons. The latter then mature into granule neurons. The development from quiescent neural stem cells to mature granule neurons in the adult SGZ involves several well-defined stages (as indicated). These newly generated neurons then migrate to the granule cell layer to integrate into the existing hippocampal circuitry. In MS, CD8+ T cells secrete Granzyme B (GrB) which affects NPCs (Type 2a and 2b cells) and inhibits their further differentiation. These NPCs also deviate from the correct path and generate astroglia instead of neurons. Finally, in some MS types, the NSCs might stay quiescent and not enter the ANG stages as reflected by the arrow pointing to the NSCs themselves.

As for hippocampus-based ANG, mounting evidence suggests that adult-born neurons may play crucial physiological roles in hippocampus-dependent functions, such as learning, memory, and mood regulation (70). The formation of these new neurons is dependent on the sub-granular zone (SGZ) of the hippocampal DG, a thin band between the granular cell layer (GCL) and the hilus. It provides a distinctive microenvironment for an adult NSC population, permitting their proliferation while also stimulating their differentiation into dentate granule neurons (Figure 3). Adult-born dentate granule neurons go through different developmental stages before they become functionally integrated into the hippocampal circuitry. Type 1 or RGLs are thought to represent the NSC population and can give rise to proliferating intermediate progenitor cells (type 2 cells) with transit amplifying characteristics, as seen in Figure 3 (71). These type 2 cells in turn generate neuroblasts (type 3 cells) that subsequently first differentiate into immature dentate granule neurons, which then migrate into the inner GCL and transform into dentate granule cells in the hippocampus (Figure 3). Within days, newborn neurons prolong dendrites toward the molecular layer and project axons through the hilus toward the CA3 (65). New neurons follow a stereotypic process for synaptic integration into the existing circuitry (72).

**Figure 4:**
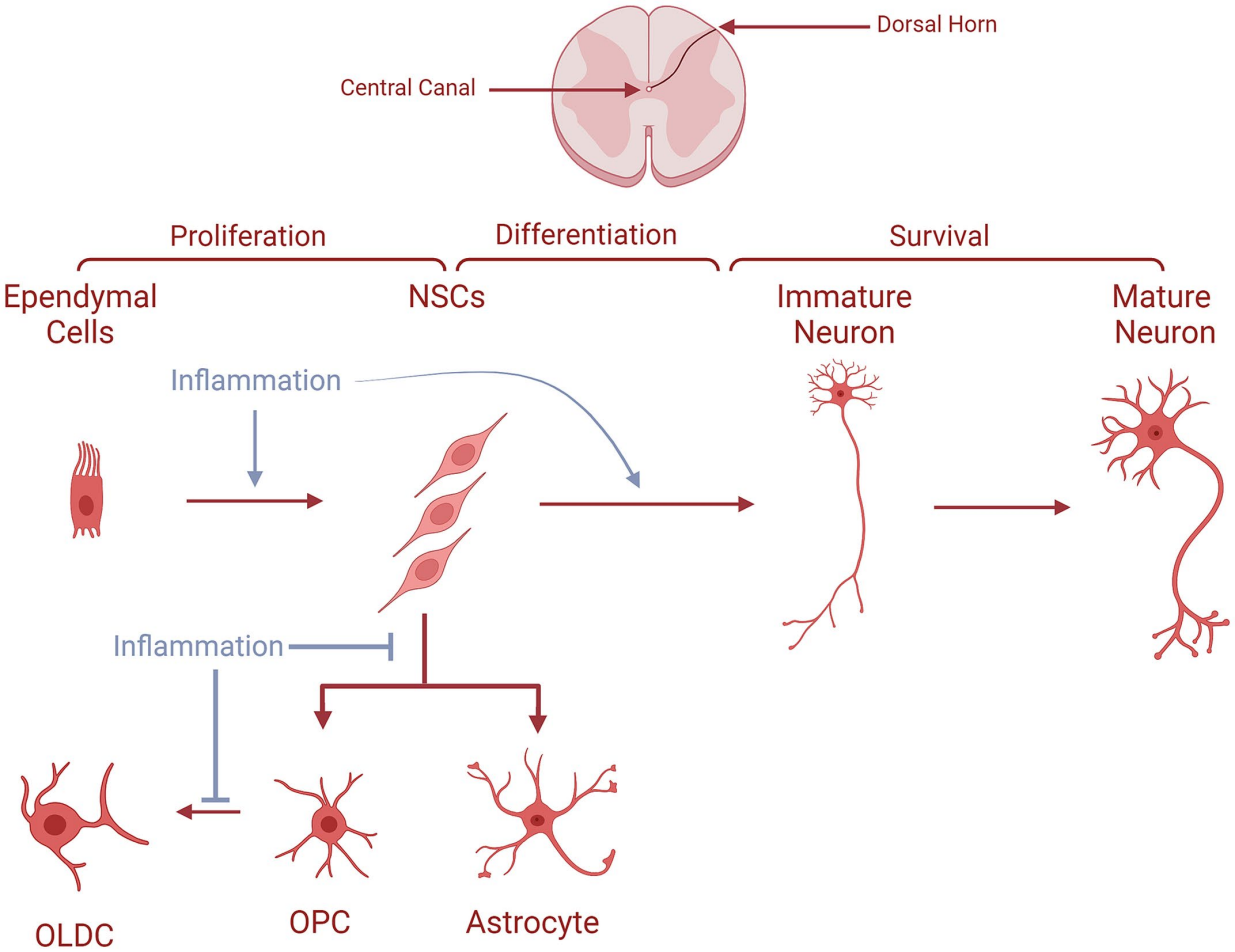
The fates of NSCs/NPCs in the spinal cord in normal conditions compared to MS. Ependymal cells proliferate and generate NSCs, which migrate along lamina IV to the superficial dorsal horn layers. During their journey, they differentiate into immature neurons becoming later mature neurons at their arrival. NSCs could also produce either astrocytes or OPCs which give rise to OLDCs. In MS, NSCs are unable to generate astrocytes and OPCs. In the case of OPC generation, they are unable to differentiate into OLCS, as indicated by the blue arrows interrupting the process.

Moreover, for the SC-based ANG, a plethora of studies have shown the presence of neurogenic niches where NPCs express neuronal properties throughout different spinal regions. In the SC, ANG naturally occurs at a constant, low rate under normal conditions, and it can be enhanced in response to pathological conditions such as disease or injury (73). The newly generated immature neurons resulting from ANG have amplified excitability and tend to migrate toward the superficial dorsal horn layers, which play a crucial role in nociceptive signaling. In steady circumstances, this process likely contributes to preserving a stable yet adaptable level of nociceptive sensitivity, reflecting a form of experience-dependent mechanism regulation parallel to other neurogenic niches. Research has also shown that SC-NSCs produced in adulthood progress through distinct differentiation stages, identifiable by specific markers like Skp2, nestin, Mash1, Ngn2, Notch3, DCX, and calretinin, ultimately maturing into neurons expressing NeuN. As seen in Figure 4, these NSCs originate in the SC ependymal layer which lines the central canal, and have the ability to undergo *in vitro* proliferation (74). Moreover, *in vivo*, the infusion of growth factors into the fourth ventricle has been shown to increase the number of BrdU^+^ cells in the central canal, with most of these cells exhibiting nestin positivity (NSC/NPC marker). Thus, ependymal cells of the central canal provide a neurogenic potential for the SC (75). These NSCs have also been shown to migrate along lamina IV, located at the medial edge of the dorsal horn, and make their way to the superficial dorsal horn layers (Figure 4). This migration process, which may extend over approximately 1 month, involves a gradual transition through all differentiation stages from neural stem cells to fully mature neurons (73). On the other hand, following SC injury (SCI), cellular proliferation was detected, specifically in the WM (76). However, these cells were largely glial progenitors that eventually differentiated into OLDCs or astrocytes. Nonetheless, under consistent stimulation following SCI, cells from the ependymal channel transformed into NPCs, which then proliferated, migrated, and differentiated into NeuN^+^ mature neurons during the acute phase of SCI (76).

Nonetheless, as stated above, gliogenesis also continues into adulthood to support functional balance under normal conditions and repairs damaged tissues under pathological conditions. This process is typically facilitated by growth factors acting via the ERK/MAPK signaling pathway (77). Furthermore, newly formed neurons stimulate undifferentiated NPCs to differentiate into various glial cells like Schwann cells, astrocytes, and OLDCs. The latter are derived from OPCs originating from multipotent NPCs (78). This transition involves stages from primitive OPCs (Olig1/2^+^/PDGFRa^−^) to mature OLDCs (oligodendrogenesis), driven by complex transcription factors and epigenetic mechanisms (79). The proneural gene Ascl plays a role in promoting OLDC differentiation in NSCs/NPCs within the hippocampus’s granular layer, a major neurogenic area in the adult brain. Astrocyte differentiation (astrogliogenesis) however mostly relies on activation of the Janus kinase (JAK)/signal transducer and activator of transcription (STAT) pathway among NSCs/NPCs progeny (80). Genetic studies with knockout mice lacking leukemia inhibitory factor (LIF), LIF receptor *β*, gp130, or STAT3 suggest that JAK–STAT signaling critically regulates astrogliogenesis in the CNS (80).

The pressure exerted by MS on the CNS milieu might affect the successful physiological transitions of NSCs/NPCs into neurons and glia. Therefore, the following sections will discuss such alterations and their consequences. However, as the majority of evidence collected around this topic originates from animal models of MS, the described data will be focused on these models rather than clinical data. This is supported by the fact that NSCs/NPCs differentiation in adult humans is still highly controversial, which limits the use of such information rigorously.

### 5.1 Immune-nervous system interaction and its impact on NSCs/NPCs fate in MS

The interaction between the immune and nervous systems plays a key role in both lesion formation and repair in MS. The progression of MS involves the complex interplay of invading immune cells, neurons, glia, and endogenous stores of NSCs/NPCs (81). However, the underlying pathways in this crosstalk are not well understood yet. Nonetheless, the fate of NSCs/NPCs is affected by their interactions with immune cells. For instance, a study by Knight et al. described the effects of murine NPCs on the three different CD4^+^ subtypes (Th1, Th2, and Th17) and vice versa (81). Their results demonstrated that the Th1 subtype is capable of inducing NPC cell death. Conversely, NPCs specifically kill pro-inflammatory Th1 and Th17 cells in a contact-dependent manner without affecting Th2 survival (81). As Th1 and Th17 cells are abundant in MS patients and corresponding animal models, specifically in zones with active neurogenesis and gliogenesis, the pool of NPCs might be at risk of depletion under these conditions.

Furthermore, Wang et al. established an *in vitro* model where they exposed human brain fetal NPCs to supernatants of resting or activated CD8^+^ T-cells isolated from healthy adult donors (82). The supernatants of the activated T-cells were able to significantly inhibit NPCs proliferation (BrdU^+^) and differentiation into neurons (*β*-III-tubulin^+^), and instead significantly enhanced their evolution into astroglia (GFAP^+^). However, these events were reversed by immunodepleting the activated T-cells’ supernatants from GrB. Therefore, these T-cells exert their inhibitory effects on neurogenesis through the secretion of GrB (Figure 3). This observation was supported by the significant decrease in NPC proliferation upon the addition of recombinant GrB into the NPC culture (82). This study also showed that GrB interacts with Giα/Go-protein-coupled receptors which then cause an elevated expression of the voltage-dependent potassium channels Kv1.3 to inhibit neurogenesis, both *in vitro* and *in vivo*. Clinical evidence further supported this claim, as GrB levels were significantly elevated in the CSF of MS patients when compared to the control subjects. Thus, GrB might be playing a crucial role in altering the fate of NPCs in MS (82).

The same research group further explored the mechanism through which certain activated microglia can enhance this neurogenic process in the WM (83). To do so, they isolated microglia from the brains of adult Sprague–Dawley rats, which were previously injected with Phosphate-buffered saline/PBS (resting) or Lipopolysaccharide/LPS (activated) to analyze resting and activated microglia, respectively. These microglia were then co-cultured in media-connected chambers with optic nerve cells (ONCs) isolated from neonatal Sprague–Dawley rats. The ONCs incubated with the activated microglia showed a significant increase in the number of neuronal cells (TUJ-1+ cells) compared to resting microglia and other controls. The majority of these TUJ-1^+^ cells originated from the macroglia of the ONC cultures (A2B5^−^ or GFAP^+^ cells) and not from the OPCs (A2B5^+^ cells), and their development was significantly accentuated by the presence of activated microglia. Moreover, a majority of TUJ-1^+^ cells generated from the co-culture with activated microglial cells were co-labeled for MAP2 and GAD65/67, signifying that these cells were mature neurons resembling GABAergic interneurons of the WM. Therefore, factors secreted by activated microglia enhance the neurogenic process from the macroglial cells and help them morph into functionally differentiated neurons (83). To help identify some of these key factors, the transcriptome and the secretome of the activated and resting microglia were analyzed, and the results pointed toward several proteins that might be involved. Among them, protease serine 2 (PRSS2), a protein whose secretion by activated microglia increased, was the most capable molecule of increasing neurogenesis (TUJ-1^+^ cells) in both monocultures of ONCs and its co-cultures with resting microglia. Whereas, secretory leukocyte protease inhibitor (SLPI), whose mRNA expression decreased in the activated microglia, was able to bring down the activated microglia-induced neurogenesis back to its baseline levels. Hence, it can be deduced that the neurogenic effect of activated microglia on OPCs, and most notably on macroglia, is mediated through the augmented secretion of PRSS2 and the lessened secretion of SLPI (83).

On the other hand, the differentiation of murine NPCs into either neurons or OLDCs was shown to be affected by microglia which are themselves influenced by inflammatory mediators (84). It was evident that the activation of microglia by IL-4 enhances the generation of neurons (*β*-III-tubulin^+^ cells). Additionally, microglia activated by IL-4 and low-dose IFN-*γ* enhanced the differentiation of NPCs into OPCs (NG2^+^ cells) and subsequently into OLDCs (GalC^+^ cells), with IL-4-activated microglia showcasing higher capacities in inducing oligodendrogenesis (84).

Hence, it is fundamental to understand the intricate interactions between various immune cell populations and NPCs/NSCs at different stages of MS to develop therapies and mitigate disease exacerbation. However, as the field of MS stem cell neuroimmunology is still in its infancy, extensive additional studies are required.

### 5.2 Alteration of NSC/NPC fates in rodent models of MS

#### 5.2.1 Alteration of NSC/NPC fates in the SVZ in rodent models of MS

In a model of EAE in C57BL/6 mice, it was observed that the normal path of SVZ-based neurogenesis was altered (85). In EAE and non-lesioned mice, similar levels of cellular proliferation occurred in the OB. However, in EAE, a significantly enhanced number of BrdU^+^ cells was detected in the SVZ, the RMS, and the CC. Additionally, in EAE, cellular proliferation was also observed in areas that are typically devoid of it in the controls, such as the Striatum (St), the Fimbria (Fi), and the Cortex (Cx). These proliferating cells even reached the demyelinated lesions of the CC and the St. The BrdU^+^ cells of the CC in the EAE animals became more enriched in neural progenitor cells or NPCs (PSA-NCAM^+^) and OPCs (NG2^+^), the latter being significantly elevated compared to the controls (85). Moreover, they followed a tracing protocol for the cells originating from the SVZ and the RMS based on staining for BrdU, CTb (Cholera toxin *β*), and DiI (1,1′-dioctadecyl-6,6′-di(4sulphopentyl)-3,3,3′,3’tetramethylindocarbocyanin) (85). They observed that the majority of these cells followed the typical path to the OB in the control animals and differentiated mostly into neurons (NeuN^+^ cells). Although in EAE, the traced cells were notably found in the OB, they irregularly migrated to the CC, the Fi, the St, and the Cx. They were also recruited into demyelinated zones within these structures, but only when the lesions existed in proximity to the SVZ. These mobilized cells were less likely to become neurons in the OB, which were substituted with GFAP^+^ astrocytes and PSA-NCAM^+^ NPCs (Figure 2). Similarly, in the CC, these recruited cells would not develop into neurons and would differentiate into NPCs, astrocytes, OPCs, and even rarely OLDCs (CNPase^+^ cells), as shown in Figure 2 (85). Therefore, it could be assumed that in MS, the normal neurogenesis process originating from the SVZ could be reprogrammed to generate OLDCs that could infiltrate and help remyelinate CNS lesions (Figure 2).

Such data were further investigated in a targeted EAE (tEAE) in which lesions and other MS characteristic symptoms could be extensively focused in the brain rather than the SC (86). This model was established in female C57BL/6 mice and showcased demyelinated lesions, axonal injury, and an abundance of inflammatory cells in the forebrains, specifically in the SVZ and the RMS. The inflammation of these neurogenic niches coincided with increased cellular proliferation (BrdU^+^) a week post-redisease induction in tEAE animals. Despite this enhanced cell division, the number of neuroblasts (DCX+) in the SVZ and RMS significantly declined compared to controls. Furthermore, these neuroblasts would exit their normal path, migrate to the St or CC, and even become apoptotic (Figure 2). Long-term observations conducted one to 2 months after disease induction, showed a persistent decline in neuroblast numbers and their dispersion into random clusters, primarily in the St. The ultrastructural analysis of the SVZ consolidated the aforementioned loss of neuroblasts and disrupted migration chains in the tEAE animals. Additionally, a significant increase of type C cells was perceived in these animals when compared to the controls (86). These findings reinforce the concept of a disrupted SVZ-based neurogenesis process in the mice.

To validate this hypothesis, NPCs (Mash1^+^) in the SVZ were investigated in more detail (86). tEAE significantly increased their number throughout the disease. However, these cells were more likely to become of the OLDC lineage (Mash1^+^ Olig2^+^) than of the neuronal lineage (Mash1^+^Dlx2^+^), as shown in Figure 2. This altered neurogenesis affected the normal structure and function of the OB where the number of newly formed mature (BrdU^+^ NeuN^+^) and immature (BrdU^+^ DCX^+^) neurons significantly decreased accompanied by a weakening of the long-term olfactory memory of the tEAE mice (86). Thus, the impaired neurogenesis led to sustained damage to the OB. However, this change at the level of the SVZ-RMS-OB path could have helped in the generation of novel OLDCs (BrdU^+^ Olig2^+^) which were then seen significantly at a later stage in the lesioned areas. This provides supporting information to the results of Picard-Riera et al. (85), which shed light on the possibility that MS could alter SVZ neurogenesis and its induced inflammation could redirect it toward the generation of OLDCs that could remyelinate the lesions (86).

Moreover, in TMEV-IDD rodents, an increase in the SVZ’s type B GFAP^+^ was found compared to controls, indicating an enhanced stem cell population. These cells differentiated into OPCs (NG2^+^), migrated to the lesioned areas, and gave rise to novel mature OLDCs (BrdU^+^ and APC^+^), potentially aiding brain remyelination (87). Thus, it could be extrapolated that in different MS rodent models, the fate of SVZ-based NSCs/NPCs could be redirected toward oligodendrogenesis in hopes of enhancing myelination and supporting the existing pool of neurons.

#### 5.2.2 Alteration of NSC/NPC fates in the hippocampus in rodent models of MS

The acute inflammatory phase of EAE significantly enhanced cellular proliferation in the SGZ of the dorsal DG of female C57BL/6 mice (88). This increased division of hippocampal cells correlated with worsening neurological capacities and elevated brain inflammation. This suggests that the newborn cells are unable to mitigate the neuropathological effects of MS, possibly because the proportion of neuroblasts (DCX^+^) among these newly formed cells (BrdU^+^) is lower in EAE animals than in the controls (88). Moreover, the percentage of astrocytes (GFAP^+^) increased significantly in EAE mice while that of mature or immature neurons (Calretinin^+^ and NeuN^+^) showcased no significant variations, among newborn cells. On the other hand, in the chronic phase of the disease, Giannakopoulou et al. found that EAE significantly increased the number of newborn cells (BrdU^+^) in the dorsal DG (89). This increase was not due to ongoing hippocampal proliferation (Ki-67 immunoreactivity) but rather to the enhanced division observed during the acute phase (88). These newly formed cells migrated more intensely to the GCL in EAE animals than in controls. Additionally, these cells were generating more neuroblasts (DCX^+^) and post-mitotic immature neurons (Calretinin^+^) but significantly fewer mature neurons (NeuN^+^) (89). Besides, more astrocytes (GFAP^+^ or S100^+^) were observed in the hippocampi of the animals affected by EAE than in the controls. Additionally, Huehnchen et al. performed experiments that further supported the aforementioned studies. In female C57BL/6 mice, EAE significantly increased cellular proliferation and newborn immature neuron and astrocyte formation in the DG, while significantly reducing the generation of new neurons during both the acute and chronic phases of the inflammation (90). Therefore, it could be hypothesized that hippocampal neurogenesis was not inhibited at its early or later stages, but that inflammation was altering it in favor of enhancing gliogenesis, as shown in Figure 3.

Molecular investigations have revealed that EAE significantly downregulates the expression levels of pro-neurogenic genes Ngn1, Ngn2, and brain-derived neurotrophic factor (BDNF) in the hippocampus. Whereas, a significant upregulation was observed for the pro-glial anti-neurogenic notch-signaling molecule Hes5 and the critical Wnt/*β*-catenin effector gene Lef1 which is responsible for NPCs proliferation in the DG. A strong increase in the expression of another member of the canonical Wnt signaling named NeuroD1 was also seen (90). These data solidify the notion that EAE-induced inflammation alters hippocampal neurogenesis, directing it more toward gliogenesis and implicating the Wnt signaling cascade in this process. Further investigating Wnt signaling’s role, Schneider et al. studied EAE in female SJL/J mice, noting a consistent increase in Wnt signaling activity in the SC and a transient rise in the hippocampus during the acute stages of the disease (around day 30). This increase in hippocampal Wnt activity was associated with significant neuronal injury and an inflammatory environment, characterized by increased Iba1^+^ and CD-68^+^ cells and elevated expression of inflammatory mediators (91). Similarly, in a passive EAE model, used to eliminate confounders associated with active immunization, hippocampal Wnt activity significantly increased only during the acute phase of the disease. Simultaneously, in the DG, a significant upregulation of the expression of several neurogenic genes and a significant rise in the number of newborn neuroblasts (BrdU^+^ DCX^+^) occurred (91). Thus, activation of Wnt signaling in the DG of these mice correlates with the *de novo* generation of hippocampal neuronal progenitors in the acute phase of EAE. Hence, this signaling might progress the initial steps of neurogenic processes, however, there is no direct indication of the generation of novel mature neurons. Yet it was shown that astrocytes were increased during the chronic phase, suggesting the divergence of the activated neurogenesis into gliogenesis (Figure 3), but further evidence is required.

On the other hand, Zhang et al. used a model of MS that allows the extensive demyelination of the hippocampus (32). During the induction period, they observed the expected hippocampal manifestations of the model: extreme demyelination, ablation of mature OLDCs (CC1^+^), and a significant reduction of OPCs (NG2^+^). These changes were reversed to control levels during the withdrawal period, except for OLDCs which remained significantly low. Moreover, the Cuprizone/Rapamycin combination decreased cellular proliferation (BrdU^+^) and the number of neuroblasts (DCX^+^) in the SGZ. Furthermore, hippocampal NSCs (Sox2^+^ GFAP^+^) became proliferatively quiescent as they expressed significantly less of the DNA replication licensing factor MCM2 in the Cuprizone/Rapamycin group than in the controls (Figure 3). This mitotic state of the NSCs most probably resulted in their reduced ability to multiply and generate neuroblasts. Although new neurons were developing in the treated animals, their dendritic lengths and spine densities significantly declined in reference to the controls. All these alterations were corrected during the withdrawal period (32). Thus, it could be hypothesized that the improvement that occurred during the remyelination stage could be the result of the hippocampal NSCs regaining their ability to proliferate and generate structurally intact functional adult-born neurons which may provide neuroprotection in MS. Therefore, in MS patients, the reactivation of such quiescent cells could open a gate to neuroprotection.

#### 5.2.3 Alteration of NSC/NPC fates in the SC in rodent models of MS

To check for the occurrence of neurogenesis in the adult SC in MS animal models, Danilov et al. established an EAE model in female Dark Agouti rats (92). Ependymal cells were thus labeled using DiI before immunization so that their progeny could be detected through the experimental timeline. In this EAE rat model (92), neuroinflammatory demyelinated lesions were detectable in the dorsal column of the SC, specifically at the thoracic and cervical levels. Lesions with complete demyelination showcased an abundance of DiI-positive cells, which were not observed in the controls’ SCs. Besides, in these lesions, neurons (NeuN^+^ and TUJ-1^+^) were observed, with a portion of them staining positively for DiI, proving that these neurons originated from the migrating ependymal cell progeny. The intralesional TUJ-1^+^ and NeuN^+^ cells were also determined to be newly generated neurons as they stained positive for BrdU and PCNA respectively, and these proliferating neuronal cells also incorporated DiI. Finally, the functional differentiation of these new neurons was validated through electrophysiological setups where they showcased their ability to fire action potentials similar to those generated by immature neurons. Hence, it can be deduced that neurogenesis of functional WM neurons can occur from ependymal cells in the adult SC to lessen the damage of spinal EAElesions (92), which could have clinical implications for MS.

This research group wanted to deepen the understanding of how adult SC-NPCs could generate functional neurons under the chronic inflammatory conditions imposed by MS. Using the EAE model in female Dark Agouti rats, they isolated SC-NPCs from different regions of the SC (cervical, thoracic, and caudal) as well as SVZ-NPCs to evaluate the change in the progeny under EAE-induced inflammation (93). SC-NPCs from the EAE animals showed an increase in the generation of neuronal cells (Ascl1^+^) and subsequently a significant rise of neurons (*β*-III-tubulin^+^) in the culture, specifically in the thoracic and caudal SC. The inflammation did not significantly affect the generation of OLDC-lineage cells (Olig2^+^). However, it caused a significant decrease in the number of OLDCs (GalC^+^) and astroglia (GFAP^+^), as seen in Figure 4. The impact of EAE on the SVZ was less noticeable on neuronal and oligodendroglial differentiation from SVZ-NPCs, while significantly improving astrogliogenesis. Therefore, it can be deduced that EAE-induced inflammation might enable SC-NPCs to generate more neurons while disabling their generation of glial cells, an event that is not observed in the SVZ, as shown in Figure 4 (93). Hence, these NPC-derived neurons might be able to migrate into MS lesions inside the SC to decrease their pathological manifestations as seen by Danilov et al. (92).

A study by Arvidsson et al. (94) further supplemented evidence that EAE impacted the neurogenic fate of NPCs from the NASC (normal-appearing SC) and the inflamed SC. NPCs derived from EAE animals had a higher *in vitro* division rate compared to NPCs derived from control animals. This enhanced proliferation was seen in the NPCs of both the inflamed SC and the NASC of the EAE rats, with more significance in the latter. The EAE-induced inflammation drastically enhanced the expression of pro-neurogenic markers in the NASC-NPC cultures compared to control NPCs: Mash-1 and Neurogenin2 at early time points and *β*-III-tubulin at later time points. Furthermore, EAE significantly decreased OLDC differentiation in the NASC-NPC culture (GalC^+^ cells). However, no significant impact was measured on the expression of the astroglial marker GFAP in either culture (94). Thus, EAE reduces OLDC generation in favor of neurogenesis in the SC, even at a distance from inflammatory sites, as seen in Figure 4. Therefore, it is possible that in MS, NPCs from both inflamed and NASC can differentiate into neurons that migrate into spinal MS lesions and alleviate them.

### 5.3 Clinical evidence of MS-induced alteration of NSC/NPC fates

Although limited, studies involving samples from MS patients have been conducted about the alteration of the fate of NSCs/NPCs under the influence of the disease. For example, Tepavčević et al. showed a significant increase in the thickness of layer II in MS patients, while the number of neuroblasts in the SVZ significantly decreased, particularly in layers II, III, and IV compared to non-neurological controls (86). Nait-Oumesmar et al. (95) confirmed these observations, showing a notable increase in cell density in layers II and III of the SVZ and elevated cellular proliferation (PCNA^+^) in MS patients’ brains. This increase in cell density may explain the thickened SVZ observed by Tepavčević et al. (86). Moreover, Nait-Oumesmar et al. further showcased that this upsurge in the number of SVZ cells could be attributed to the significant elevation in the numbers of progenitors (PSA-NCAM^+^) and astrocytes (GFAP^+^) (95). These cells were seen migrating along or away from the SVZ and co-expressing the OPC marker Sox9. Besides, some cells also co-expressed OPC markers Sox10 or Olig2 and NG2, suggesting that in MS, SVZ proliferating cells generate fewer neuroblasts and more OLDC-lineage cells. Furthermore, Nait-Oumesmar et al. identified round-shaped progenitor cells expressing only PSA-NCAM, not GFAP, in the lesions and surrounding WM of the MS patients (95). These progenitors were less prevalent in chronic silent lesions and Normal Appearing White Matter (NAWM) than in active and chronic lesions. PSA-NCAM^+^ progenitors were more abundant in active lesions near the SVZ, retaining the ability to proliferate and generate OPCs (positive for Sox10 or Olig2 and NG2) and OLDCs. Thus, SVZ progenitors could migrate to the MS lesions where they would generate OLDC lineage cells that could help remyelinate the brain. This process could be related to altered neurogenesis as the PSA-NCAM^+^ progenitors would rarely give rise to neurons (*β*-III-tubulin^+^) in the lesions (95).

The Marburg Variant of MS, a severe and rapidly progressing form described by Otto Marburg in 1906, involves large, detectable lesions in the brain’s WM, leading to rapid decline and often resulting in death within weeks. Neuropathological features include extensive demyelination, loss of oligodendrocytes, WM necrosis, and immune cell infiltration, while axons remain intact. Postmortem brain analysis of a Marbug variant patient showed an increased thickness of layers II and III of the SVZ compared to the non-neurological control (96). Nonetheless, cellular proliferation (Ki-67 immunoreactivity) was almost completely absent in the SVZ of the MS patient. Moreover, GFAPδ and Sox2 presented a dramatically low expression in the SVZ of the patient compared to the control, signifying a depletion of NSCs. Likewise, glial progenitor cells (Musashi^+^) were absent, and NPCs were scarce (PAX6^+^). These variations were extremely discrepant when compared to the control subject, thus demonstrating a strong inhibition of neurogenesis in the Marburg variant of MS in the SVZ. This intense inhibition could be accredited to the exacerbated inflammation in such patients. However, some OLDC generation (NG2^+^) was seen in the lesioned CC (96). This last observation could provide insight into the fact that although neurogenesis was almost abolished, its remnants were directed toward the generation of OLDCs in the hope of diminishing the damage MS caused to the brain. Similar to the SVZ, in the hippocampus of the same patient, cellular proliferation, NSCs, NPCs, and glial progenitor cells were extremely limited when compared to the control. These findings show the almost complete abolishment of hippocampal neurogenesis in such cases (96).

Additionally, a postmortem study by Chang et al. evaluated whether neurogenesis could occur in demyelinated subcortical WM of MS patients (97). They evaluated this process in demyelinated lesions that were either acute or chronic and NAWM from both MS patients and control individuals. In the latter specimens, no differences in the number and morphology of the WM neurons were observed. However, neurons (MAP2^+^ or NeuN^+^ cells) were rarely detected in acute demyelination zones and most chronic lesions. Nonetheless, the number of neurons significantly increased in 15/59 of the chronic lesions compared to the surrounding NAWM. Thus, both mature (MAP2^+^/NeuN^+^) and immature (MAP2^−^/NeuN^+^) neurons can exist in this subset of chronic lesions in MS brains. The mature neurons also showed functional differentiation through their expression of various WM neuron markers. Moreover, in these neuron-rich lesions, the synaptic density increased dramatically compared to the NAWM. Additionally, these lesions contained a relatively elevated number of MHC-II^+^ cells compared to active and other chronic lesions, while also demonstrating a distinct morphology from their immune cells. This morphologically distinct population of activated microglia might be favoring the neurogenesis seen in this subset of chronic regions as they might enable a rather enriching and non-destructive niche that allows the *de novo* development of WM neurons (97). It was also shown that the SVZ bordering the WM lesions had significantly more NeuN^+^ cells than the SVZ neighboring the NAWM in MS patients. Most of these cells also expressed Dlx2, a transcription factor expressed by progenitor cells committed to interneuron production (97). Therefore, it could be assumed that the SVZ would be able to provide a source for new neurons in MS patients, whose survival and differentiation in the demyelinated WM might be enhanced by the presence of a distinct site of activated microglia present at the lesion sites.

## 6 Novel MS treatments based on NSCs/NPCs fate alteration

The current clinically adopted treatments for MS focus on limiting disease progression and symptom control. However, no current treatments center around the reversal of the disease process, which is a vital area for research and development. Hence, treatments that empower neurogenesis and gliogenesis have recently surged as promising MS therapies that hold the potential to go beyond disease control and abolish life-disabling manifestations. Such treatments are mainly divided into chemical agents and stem cell therapies, both of which will be briefly described in the following sections. We will only focus on a limited number of examples for each treatment subtype to highlight the relevance of these unorthodox approaches in changing the fates of NSCs/NPCs in the MS-altered CNS milieu to remediate the pathophysiology of this disease.

### 6.1 Chemical agents

Many compounds are continuously trialed as potential MS cures. Nevertheless, only a few molecules hold the capacity to modify neurogenesis and gliogenesis in order to eradicate MS symptoms. Examples of these drugs include repurposed well-established clinically utilized drugs, which offer safe medical options. Whereas, other molecules inspired by folk medicine and medicinal plants, which can possess incomparable potencies with understudied clinical relevance and security. Moreover, since these natural products are scarce, the discovery of novel small molecules from chemical libraries with such capacities has also been a hot topic in the context of MS therapeutics. Finally, the exploitation of the activation or inhibition of endogenous molecules to manipulate neurogenesis/gliogenesis has also been brought into question. Examples of these various interventions are described below and can be seen in Table 1.

**Table 1:** Novel MS therapies influencing neurogenesis or gliogenesis.

Treatment	Model	Effects on MS	Effects on neurogenesis	Target	Reference
Intraperitoneal injection of Polyprenols (12 mg/kg) for 5 weeks	Cuprizone-induced demyelination model in male CD-1 mice	Significant decrease in demyelination in the brain Significant increase in locomotor activity and elimination of anxiety-like behaviors	Significant decrease in oligodendrogenesis and significant increase in neurogenesis in the SVZ and the DG	N/A	(105)
Addition of Omeprazole (2.5, 5, 10, 20 μM)	Primary OPCs isolated from newborn Sprague Dawley rats	Increased expression of MBP in a dose-dependent manner Up-regulated expression of genes related to remyelination (CNP, PLP, MAG)	Increase in differentiation and maturation of OLDC lineage	Activation of ERK1/2 and p38 MAPK	(98)
Intraperitoneal injection of Omeprazole (10 mg/kg) given every other day for 2 weeks	Cuprizone-induced demyelination model in male C57BL/6 mice	Improvement of the impaired motor coordinative function of demyelinated mice Increase in myelinated axons in the CC Increased expression of MBP and CNPase	Increase in OPC numbers, differentiation, and maturation	N/A	
Leonurine (5 μM) for 5 days	OPCs differentiated from NSCs of the embryonic murine Cx	Increase in MBP and PLP expression	Enhanced OPC differentiation to OLDCs	Inhibition of H3K27 methylation through the increase in JMJD3 expression	(104)
Leonurine (5 μM) for 2 days	Cerebellar slice cultures from newborn mice	Increase in MBP expression	N/A	N/A	
Intraperitoneal injection of Leonurine (60 mg/kg)	EAE model in male C57BL/6 mice	Alleviation of clinical severity Reduction in CNS inflammation and myelin damage Inhibited infiltration of pathogenic autoimmune T cells into the CNS Suppression of local neuroinflammation Increased expression of MBP and PLP in CNS Enhanced remyelination in the CNS	Enhanced OPC differentiation into mature OLDCs in the inflammatory loci of the CNS	N/A	
	Cuprizone-induced demyelination mouse model in female C57BL/6 mice	Enhanced spontaneous remyelination, especially in the CC	Enhanced OPC differentiation into mature OLDCs in the CC	N/A	
Subcutaneous injections of GA (2 mg/mouse) for 5 to 8 days	EAE model in female C57BL/6 mice	Reduction in the neuronal/axonal damage in the brain Decrease in activation of microglia in the brain	Increase in NPCs proliferation in SVZ, SGZ, and GCL Increase in SVZ - NPCs migration to the RMS Migration of the SVZ-NPCs to the LCS Increase in NPCs migration to lesion sites Increase in NPCs differentiation into NeuN+ cells at the lesion sites enabling neuronal regeneration	Increase in BDNF expression	(99)
Addition of 10 μg/mL of GA to T cells for 3 days	Embryonic brain-derived forebrain cells in culture exposed to GA-reactive T cell condition media	N/A	Increase in OPC numbers and cellular proliferation	Increase in IGF-1 secretion by T cells	(100)
Daily subcutaneous injection with GA (2 mg per mouse) for 7 days	Lysolecithin-induced demyelination of the spinal cord in male C57BL/6 mice	Increase in spinal cord remyelination	Increase in SC OPC numbers	Increase in IGF-1 and BDNF expression	
Addition of Thymosin Beta 4 (25, 50 ng/mL) for 24 h	N20.1 premature OLDC cell line	N/A	Increase in OPC proliferation in a dose-dependent manner	N/A	(110)
Intraperitoneal injections of Thymosin beta 4 (6 mg/kg) every 3 days, a total of 5 doses	EAE model in female SJL/J mice	Delay in EAE onset Decrease in severity of disease Decrease in inflammatory infiltrates in the brain	Increase in OPCs and mature OLDCs in the brain Increase in OPC proliferation and differentiations into OLDCs	N/A	
Addition of 1 μM of U-50488 for 3 days	OPC culture from postnatal murine cortices	N/A	Increase in differentiation of OPCs into mature myelinating OLDCs	Kappa-opioid receptor agonist	(106)
Addition of 1 μM of U-50488 for 5 days	Rat OPC and rat dorsal root ganglion neurons co-culture	Enhanced myelination			
Oral gavage of U-50488 (10 mg/kg/day) for 10 days	Lysolecithin-induced focal CC demyelination mouse model	Acceleration of remyelination in lesions			
Addition of U-50488 at 1 μM for 10 days	Human induced pluripotent stem cell-derived OPCs	N/A			
Anti-EphrinB3 antibodies	Primary OPC culture from neonatal Sprague Dawley rats	Increase in MBP expression	Enhanced OPC differentiation	Neutralization of EphrinB3	(109)
Anti-EphrinB3 antibodies were given via an osmotic pump (200 μg/mL) for 11 days	Rat model of remyelination in female Sprague Dawley rats by bilateral stereotactic injection of ethidium bromide into the caudal cerebellar peduncle	Increase in CNS remyelination			
Intraperitoneal injection of Xyloside (2.4 mg/animal/day) for 10 days	Theiler’s murine encephalomyelitis virus-induced demyelinating disease in female SJL/J mice	Improvement of both horizontal and vertical motor deficits Reduction of astrocyte activation	N/A	Reduction of CSPGs in lesions	(107)
Intraperitoneal injection of UCM03025 (5 mg/kg/day) for 10 days		Improvement of both horizontal and vertical motor deficits Reduction of astrocyte activation Reduction of immune cell infiltration into the SC, microglia activation, and neuroinflammation Improvement in axonal integrity Enhanced remyelination	Increase in OPC numbers, proliferation, and differentiation into OLDCs at the lesion sites	Inhibition of 2-AG hydrolysis	

#### 6.1.1 Clinically utilized medications

Omeprazole is a proton pump inhibitor, traditionally used for the treatment of gastric ulcers. In a study investigating Omeprazole’s impact on primary OPCs from newborn rats, the drug showed promising results (Table 1). Omeprazole dose-dependently increased MBP expression while also upregulating genes associated with myelin repair (CNP, PLP, MAG). It also stimulated the OPCs’ maturation and differentiation, which might have enhanced myelin production. Omeprazole exerted its action on OPCs through the activation of ERK1/2 and p38 MAPK cell signaling pathways (98). The *in vitro* results prompted the authors to further investigate Omeprazole’s effects *in vivo*. In a cuprizone-induced demyelination model in male C57BL/6 mice, Omeprazole improved impaired motor coordinative function. The drug also increased the number of myelinated axons particularly in the corpus callosum (CC), as well as the expression of MBP and CNPase. These effects could be attributed to the observed increase in OPC numbers, differentiation, and maturation *in vivo* (98). Thus, Omeprazole could serve as a treatment for MS through its stimulation of oligodendrogenesis.

Glatiramer Acetate (GA), an approved treatment for MS, was studied in an EAE model in female C57BL/6 mice to determine its exact effects on MS hallmarks (Table 1). A reduction in the neuronal/axonal damage concomitantly with a decrease in activation of microglia in the brain was evident (99). Moreover, GA increased NPCs proliferation in SVZ, SGZ, and GCL. Additionally, there was an increase in the typical migration of SVZ - NPCs to the RMS and the creation of a migratory path to the LCS. NPCs migration to lesion sites also increased upon treatment with GA. At the lesion sites, NPCs differentiated into NeuN^+^ cells more notably after GA administration. GA treatment led to an increase in the expression of BDNF (99). BDNF is a vital protein for neuronal growth and survival, suggesting a supportive environment for brain repair and neurogenesis. GA also impacted oligodendrogenesis in a lysolecithin-induced demyelination mouse model. The drug caused an increase in OPC numbers enabling an upsurge in SC remyelination (100). GA exercised its action on OPC presence and cellular proliferation by shifting T-cells to a Th2 phenotype as evidenced by the *in vitro* results. These GA-reactive T-cells generate many molecules that can impact OPC behavior *in vitro* and *in vivo* such as IGF-1 and BDNF (100). Thus, GA continues to be a promising drug for treating progressive MS through its impact on both neurogenesis and oligodendrogenesis.

Treatment of MS with IFN-*β* has been shown to reduce the progression of disability and hinder brain atrophy, suggesting that IFN-β acts directly on the CNS. It was the first disease-modifying therapy for MS, offering patients a treatment that lowers relapse rates and delays disability onset. Currently, four IFN-β medications are approved for treating relapsing forms of MS: subcutaneous (SC) IFNβ-1b, SC IFNβ-1a, intramuscular IFNβ-1a, and the recently approved SC peginterferon beta-1a (101). Moreover, a study tested whether IFN-β acts directly on the CNS by promoting NPC survival and/or proliferation in MS (102). Results suggested that IFN-β played a direct neuroprotective role by promoting the survival of mouse NPCs at low concentrations *in vitro* (1 and 10 ng/mL) while demonstrating no influence on activating oligodendrocyte differentiation (102). Another study demonstrated for the first time that IFN-β-1b directly affects human NPCs *in vitro* (103). The results of this study indicated that IFN-β promotes NPC proliferation, enhances differentiation in a concentration-dependent manner, and protects against apoptosis (103). These data suggest that IFN-β treatment can function not only as an immune modulator but also as an enhancer of NPCs.

#### 6.1.2 Plant-based therapies

Leonurine is an alkaloid extracted from traditional Chinese medicine *Herba leonuri* was investigated for its impact on oligodendrogenesis *in vitro* and *in vivo*, as shown in Table 1 (104). In OPCs differentiated from NSCs of the embryonic murine Cx, an increase in MBP and PLP expression was noted. In addition, Leonurine enhanced OPC differentiation to OLDCs via the increase in the expression of the demethylase JMJD3 which consequently inhibited Histone 3 Lysine 27 methylation (104). Similarly, in the cerebellar slice cultures from newborn mice, an increase in MBP expression was evident after Leonurine treatment (104). To further evaluate the effects of Leonurine in MS animal models, 2 models were established: the EAE model in male C57BL/6 mice and the cuprizone-induced demyelination mouse model in female C57BL/6 mice. In the latter, particularly in the CC, enhanced spontaneous remyelination and enhanced OPC differentiation into mature OLDCs were marked post-Leonurine treatment (104). In the EAE model, the effects of Leonurine on MS were numerous. Most importantly, the drug alleviated the clinical severity of the disease and reduced CNS inflammation and myelin damage. Additionally, an important increase in the expression of MBP and PLP in the CNS was seen, leading to enhanced remyelination in the CNS. OPCs in the EAE model differentiated and matured similarly to the cuprizone-induced demyelination model, with a focus on the inflammatory loci of the CNS (104). Thus, it could be extrapolated that Leonurine could limit MS, mainly through its enhancement of oligodendrogenesis and subsequently remyelination.

Polyprenols are plant-based long-chain isoprenoid alcohols, that have been linked to neuroprotective, immunomodulating, and proliferative activities. Thus, Polyprenols from *Picea abies (L.) Karst* were studied in a cuprizone-induced demyelination model in CD-1 mice, as seen in Table 1 (105). A significant decrease in the demyelination in the brain was seen accompanied by a significant increase in locomotor activity and elimination of anxiety-like behaviors in the Polyprenols-treated mice. Moreover, a significant decrease in oligodendrogenesis was noted as well as a significant increase in neurogenesis in the SVZ and the DG in the treated mice (105). Hence, Polyprenols could potentially be utilized to alleviate MS-related behaviors through its upregulation of neurogenesis. Consequently, both Leonurine and Polyprenols can be considered favorable therapies in MS treatment, upon further evaluation of their clinical relevance and safety.

#### 6.1.3 Discovery of novel molecules

Small molecules were either discovered or developed from pre-existing chemical skeletons to combat MS. For the *de novo* discovery, upon screening a library of small molecules with G-protein-coupled receptor capacity, the compound (±)U-50488 was discovered, as shown in Tabe 1 (106). This molecule is a Kappa-opioid receptor agonist that increased the differentiation of OPCs into mature myelinating OLDCs in cultures of post-natal murine Cx OPCs and human induced pluripotent stem cells-derived OPCs (106). Similar actions were also observed in a co-culture model of rat OPCs and rat dorsal root ganglion neurons where enhanced myelination was also noted. Furthermore, (±)U-50488 accelerated the remyelination of lesions in a lysolecithin-induced focal CC demyelination mouse model, through the increase of oligodendrogenesis (106).

Whereas, for drug development, UCM03025 is a prime example (Table 1). Since in the TMEV-IDD murine model, astrocyte activation and chondroitin sulfate proteoglycans (CSPGs) accumulation occurred in the lesions, Xyloside, an inhibitor of CSPGs synthesis was trialed as a treatment (107). This drug improved motor deficits and reduced astrocyte activation. As CSPG production is modulated by 2-Arachidonoylglycerol (2-AG), the inhibition of its hydrolysis via a potent reversible inhibitor of its lipase was explored in TMEV-IDD. UCM03025 was previously developed by the same research group (108) as an MS treatment. UCM03025 administration caused similar changes to Xyloside while also reducing immune cell infiltration into the SC, microglia activation, and neuroinflammation. UCM03025 further improved axonal integrity and enhanced remyelination. These actions might be largely based on its ability to increase OPC numbers, proliferation, and differentiation at lesion sites (107). Thus, both of these experimental drugs showcase MS-healing abilities through their pro-oligodendrogenesis actions.

#### 6.1.4 Exploitation of endogenous molecules

Bioactive molecules found within the organism can deeply impact neurogenesis and gliogenesis. For instance, Ephrin B3 is a myelin-associated protein with the ability to inhibit OPC differentiation. The neutralization of this protein by specific antibodies thus allowed the enhancement of OPC differentiation both *in vitro* and *in vivo*, as observed in Table 1 (109). Other effects of the neutralizing antibodies included the increase in MBP expression in primary neonatal rat OPC cultures and the rise in CNS remyelination in a rat model (109).

Moreover, Thymosin Beta4 is an evolutionarily conserved protein known mainly as an actin polymerization regulator, with well-established effects on cell motility and organogenesis. Thymosin Beta4 was looked into in an N20.1 premature OLDC cell line and found to induce OPC proliferation in a dose-dependent manner (110). Additionally, an increase in OPCs and OLDCs in the brain was seen in the EAE mouse model, through enhanced proliferation and differentiation of the OPCs. These changes in oligodendrogenesis were complemented by a delay in EAE onset, a decrease in disease severity, and inflammatory infiltrates in the brain (110).

The aforementioned remedies hold tremendous potential for clinical application and translation into MS-targeted therapies. Further research is indicated to establish their safety and dose-related regimens with the potential of combining various therapies.

### 6.2 Stem cells

Stem cells (STCs) have been extensively studied as tools to mediate the pathological aspects of many neurodegenerative and demyelinating malignancies. For MS, many tactics that rely on STCs and the subsequent changes in neurogenesis and gliogenesis have been developed. Their efficiency has been extensively demonstrated in multiple experimental models of MS. For instance, upon peritoneal administration, both allogeneic and syngeneic mice bone marrow mesenchymal stem cells (BM-MSCs) reduced disease severity and neuropathology in a murine EAE model (111). Moreover, human BM-MSCs demonstrated therapeutic effects in both chronic and relapsing–remitting murine models of EAE, even at the peak of the disease, when they were injected subcutaneously (112). These MSCs also reduced multiple hallmarks of MS such as inflammatory cell infiltration into the CNS and extensive demyelination. This effect might be exerted through the migration of the BM-MSCs to the demyelinated regions where they enabled oligodendrogenesis and reduced astrogliogenesis. They also caused a shift from a myelin-specific Th1 cells-dominated immune milieu to an anti-inflammatory Th2-dominated one (112). The effects of BM-MSCs might have been mediated by secreted molecules, as their condition media (CM) significantly elevated the proportions of OPCs, OLDCs, and neurons in a cortical neurosphere culture from newborn mice (113). Additionally, modifying human BM-MSCs to differentiate into neurotrophic factor-secreting cells (NFTCs) could enhance their efficacy against MS. NFTCs showed greater potency than their precursor BM-MSCs in improving clinical scores and survival rates when injected intracerebroventricularly into EAE mice (114). These NFTCs resided around the lateral ventricles and secreted BDNF *in vivo*. This molecule among others secreted *in vitro* by the NFTCs might explain their added capacities in MS, as they might stimulate the EnSTC population (114).

Another STC type that is looked into for MS therapies is adipose-derived stem cells (ADSCs). In a chronic EAE mouse model, intravenously administered murine ADSCs reduced the clinical severity of the disease when used either as a preventive measure or a therapeutic intervention (115). Notably, ADSCs were more effective than BM-MSCs in therapeutic applications, providing superior neuroprotection and significantly reducing inflammation and demyelination (115). ADSCs impacted T-cell proliferation and cytokine production and resulted in a shift in the T-cell activation toward a Th2 phenotype. Moreover, ADSCs migrated to CNS lesions where they differentiated to OPCs or endorsed the endogenous OPC population through secreting growth factors like BDNF (115). Like BM-MSCs, ADSCs could be differentiated under culture conditions into NFTCs. Razavi et al. employed this approach by injecting a combination of ADSCs and their NFTC progeny intralesionally into a rat demyelination model (116). This dual treatment alleviated clinical signs and nearly restored the myelination status of the SC, outperforming ADSC monotherapy. The combination therapy also increased both mature and immature OLDCs at the lesion sites (116). Systemic injections of human ADSCs in combination with Pregnenolone, a pleiotropic neurosteroid, were also used to treat demyelination caused by cuprizone in rats (117). This combination of Pregnenolone/ADSCs significantly enhanced remyelination in the CC and improved the animals’ performance in the basket test, surpassing the effects of standalone treatments (117). The same research group further explored the impact of this combination in the same model, while changing the injection site of the ADSCs from the bloodstream to the ventricles. This modification further enhanced the effects of this combination when addressing the aforementioned criteria (118).

On another note, Clark et al. used STCs from the human placenta as EAE treatments (119). Either intervention remarkably improved motor function outcomes and reduced DNA damage in oligodendroglia, thus decreasing their death while enhancing the generation of mature OLDCS. This allowed for an enhanced myelination of the SC (119). These STCs might have also exerted neuroprotection *in vivo* through the secretion of growth factors like BDNF and HGF (Hepatocyte Growth Factor), as this would be in accordance with their *in vitro* capacities (119). Brown et al. also used MSCs from the human umbilical cord and fetal placenta to treat EAE, by first differentiating them into NSCs capable of generating neurons and OLDCs *in vitro* (120). Then, injecting these NSCs or MSCs into the tail veins of diseased animals improved neurobehavior and disease scores, with NSCs yielding better outcomes (120). NSCs were more efficient at CNS homing, survival, decreasing immune infiltration, and creating an anti-inflammatory environment. They specifically reduced the pool of Tregs and Th17 cells in the CNS, which may help regulate CNS autoreactive T-cells (120). NSCs could have provoked this through their secretion and potentially the downstream activation of the Endogenous STCs pool. Additionally, NSCs significantly improved myelination in EAE animals by differentiating into neuronal and oligodendroglial cell lineages, enhancing myelin production and neuroprotection (120). Besides, Shu et al. utilized human amnion mesenchymal cells (AMCs) to treat EAE established in C57BL/6 mice (121). The AMCs lessened disease severity and the pathological hallmarks of EAE. AMCs inhibited the production of pro-inflammatory cytokines and autoreactive T-cell presence in the CNS. AMCs also secreted a plethora of growth factors that could trigger the EnSTCs and thus promote myelination and repair (121).

Additionally, human olfactory mucosal mesenchymal stromal cells (OM-MSCs) were used to treat EAE in a C57Bl/6 J mouse model and their effects were compared to human BM-MSCs (122). Both MSC types ameliorated disease severity if delivered during the initial onset of symptoms. Yet, only OM-MSCs improved outcomes when administered at the disease peak, demonstrating their superior capacity for treating EAE. OM-MSCs showed enhanced abilities to reduce inflammatory cell recruitment, decrease BBB disruption, and promote axonal survival (122). Despite localizing only to blood vessels and not the CNS, OM-MSCs exerted systemic effects, significantly lowering levels of circulating and spinal IL-16 and reducing immune cells’ capacity to synthesize this cytokine. The role of IL-16 was further verified *in vitro* in both rat OPC and SC cultures (122). OM-MSCs’ superior effects compared to BM-MSCs may be attributed to their additional impact on IL-16 signaling.

These studies among others showcase the ability of STCs as viable therapies for MS and showcase their undeniable potential especially when administered solo or with various adjuvant chemical agents. Evidently, more clinical data are needed to verify their safe and efficient use in MS patients. However, promising results are being collected and can be seen in detail by Cecerska-Heryć et al. (123).

## 7 Conclusion

In summary, MS is a convoluted disease with profound impacts on both the nervous and immune systems. This review shows the importance of understanding the underlying mechanism and clinical manifestation of this disease. It also underscores the role of NSCs and NPCs in the course of MS by promoting neurogenesis or gliogenesis. Thus, these cellular populations can in fact alter their differentiation fates in MS to try and alleviate the burden of this disease. Moreover, treatments that are able to favor such actions should be taken into consideration as efficient MS therapies. However, most of the data used to make this assumption originated from animals. Therefore, more clinical studies are required to strengthen knowledge about the involvement of NSCs/NPCs in the pathophysiology of MS and the ability of drugs affecting their fates to be used as clinical candidates.

## Glossary

AE: 2-AG: 2-Arachidonoylglycerol
ADSCs: Adipose-Derived Stem Cells
AMCs: Amnion Mesenchymal Cells
ANG: Adult Neurogenesis
APC: Antigen Presenting Cell
Ascl1 or Mash1: Achaete-scute homolog 1
BBB: Blood Brain Barrier
BDNF: Brain-Derived Neurotrophic Factor
BM-MSCs: Bone Marrow Mesenchymal Stem Cells
BrdU: Bromodeoxyuridine
CC: Corpus Callosum
CIS: Clinically Isolated Syndrome
CM: Condition Media
CNPase: 2′,3’-Cyclic-nucleotide 3′-phosphodiesterase
CNS: Central Nervous System
CSF: Cerebrospinal Fluid
CSPG: Chondroitin Sulfate Proteoglycans
CTb: Cholera Toxin *β*
Cx: Cortex
DCX: Doublecortin
DG: Dentate Gyrus
DiI: 1,1′-dioctadecyl-6,6′-di(4sulphopentyl)-3,3,3′,3’tetramethylindocarbocyanin
DNA: Deoxyribonucleic Acid
EAE: Experimental Autoimmune Encephalomyelitis
Fi: Fimbria
FoxP3: Forkhead box protein 3
GA: Glatiramer Acetate
GAD65/67: Glutamate Decarboxylase 65/67
GalC: Galactosylceramidase
GCL: Granular Cell Layer
GDNF: Glial cell-derived Neurotrophic Factor
GFAP: Glial Fibrillary Acid Protein
GM-CSF: Granulocyte-Macrophage Colony-Stimulating factor
GrB: Granzyme B
HGF: Hepatocyte Growth Factor
IFN: Interferon
IL: Interleukin
LPS: Lipopolysaccharide
MAP 2: Microtubule-Associated Protein 2
MBP: Myelin Basic Protein
MgTX: Margatoxin
MHC: Major Histocompatibility Complex
MOG: Myelin Oligodendrocyte Glycoprotein
MRI: Magnetic Resonance Imaging
mRNA: Messenger Ribonucleic Acid
MS: Multiple Sclerosis
NASC: Normal Appearing Spinal Cord
NAWM: Normal Appearing White Matter
NeuN: Neuronal Nuclear Antigen
NFTCs: Neurotrophic Factors Secreting Cells
NG2: Neuron-Glial Antigen 2
NGF: Nerve Growth Factor
NK: Natural Killer
NO: Nitric Oxide
NPC: Neural Progenitor Cell
NSC: Neural Stem Cell
OB: Olfactory Bulb
OCB: Oligoclonal Bands
OLDC: Oligodendrocyte
Olig2: Oligodendrocyte transcription factor
OM-MSCs: Olfactory Mucosal Mesenchymal Stem Cells
ONCs: Optic Nerve Cells
OPC: Oligodendrocyte Progenitor Cell
PBS: Phosphate-buffered saline
PCNA: Proliferating Cell Nuclear Antigen
PLP: Proteolipid Protein
PPMS: Primary Progressive Multiple Sclerosis
PRSS2: Protease Serine 2
PSA-NCAM: Polysialylated Neuronal Cell Adhesion Molecule
RGL: Radial Glia-Like cell
RMS: Rostral Migratory Stream
RNA: Ribonucleic Acid
RNS: Reactive Nitrogen Species
ROS: Reactive Oxygen Species
RRMS: Relapsing–Remitting Multiple Sclerosis
SC: Spinal Cord
SCI: Spinal Cord Injury
SDF1: Stromal cell-derived Factor 1
SGZ: Sub-granular Zone
SLPI: Secretory Leukocyte Protease Inhibitor
SPMS: Secondary Progressive Multiple Sclerosis
STCs: Stem Cells
St: Striatum
SVZ: Subventricular Zone
TAP: Transit Amplifying Cell
tEAE: Targeted Experimental Autoimmune Encephalomyelitis
TMEV-IDD: Theiler’s Murine Encephalomyelitis Virus-Induced Demyelinating Disease
TNF-*α*: Tumor Necrosis Factor Alpha
Tregs: Regulatory T Cells
TUJ-1 or *β*-III-Tubulin: Class III β-Tubulin
WM: White Matter

## References

[1] Travers BS Tsang BKT Barton JL. Multiple sclerosis: diagnosis, disease-modifying therapy and prognosis. Aust J Gen Pract. (2022) 51:199–206. doi: 10.31128/AJGP-07-21-6103 35362004

[2] Zindler E Zipp F. Neuronal injury in chronic CNS inflammation. Best Pract Res Clin Anaesthesiol. (2010) 24:551–62. doi: 10.1016/j.bpa.2010.11.001 21619866

[3] Wallin MT Culpepper WJ Campbell JD Nelson LM Langer-Gould A Marrie RA. The prevalence of MS in the United States: a population-based estimate using health claims data. Neurology. (2019) 92:e1029–40. doi: 10.1212/WNL.0000000000007035, PMID: 30770430 PMC6442006

[4] Dighriri IM Aldalbahi AA Albeladi F Tahiri AA Kinani EM Almohsen RA. An overview of the history, pathophysiology, and pharmacological interventions of multiple sclerosis. Cureus. (2023) 15:e33242. doi: 10.7759/cureus.33242 36733554 PMC9888604

[5] Dobson R Giovannoni G. Multiple sclerosis – a review. Eur J Neurol. (2019) 26:27–40. doi: 10.1111/ene.13819 30300457

[6] Ward M Goldman MD. Epidemiology and pathophysiology of multiple sclerosis. Continuum. (2022) 28:988–1005. doi: 10.1212/CON.0000000000001136 35938654

[7] Orton SM Herrera BM Yee IM Valdar W Ramagopalan SV Sadovnick AD. Sex ratio of multiple sclerosis in Canada: a longitudinal study. Lancet Neurol. (2006) 5:932–6. doi: 10.1016/S1474-4422(06)70581-6, PMID: 17052660

[8] Sintzel MB Rametta M Reder AT. Vitamin D and multiple sclerosis: a comprehensive review. Neurol Ther. (2018) 7:59–85. doi: 10.1007/s40120-017-0086-4, PMID: 29243029 PMC5990512

[9] Napier MD Poole C Satten GA Ashley-Koch A Marrie RA Williamson DM. Heavy metals, organic solvents, and multiple sclerosis: an exploratory look at gene-environment interactions. Arch Environ Occup Health. (2016) 71:26–34. doi: 10.1080/19338244.2014.937381, PMID: 25137520 PMC4334728

[10] Handel AE Williamson AJ Disanto G Dobson R Giovannoni G Ramagopalan SV. Smoking and multiple sclerosis: an updated Meta-analysis. Jacobson S, editor. PLoS One. (2011) 6:e16149. doi: 10.1371/journal.pone.0016149 21249154 PMC3020969

[11] Hollenbach JA Oksenberg JR. The immunogenetics of multiple sclerosis: a comprehensive review. J Autoimmun. (2015) 64:13–25. doi: 10.1016/j.jaut.2015.06.010 26142251 PMC4687745

[12] The International Multiple Sclerosis Genetics Consortium. Class II HLA interactions modulate genetic risk for multiple sclerosis. Nat Genet. (2015) 47:1107–13. doi: 10.1038/ng.3395, PMID: 26343388 PMC4874245

[13] Capasso N Virgilio E Covelli A Giovannini B Foschi M Montini F. Aging in multiple sclerosis: from childhood to old age, etiopathogenesis, and unmet needs: a narrative review. Front Neurol. (2023) 14:1207617. doi: 10.3389/fneur.2023.1207617, PMID: 37332984 PMC10272733

[14] Balint B Haas J Schwarz A Jarius S Fürwentsches A Engelhardt K. T-cell homeostasis in pediatric multiple sclerosis: old cells in young patients. Neurology. (2013) 81:784–92. doi: 10.1212/WNL.0b013e3182a2ce0e, PMID: 23911752

[15] Tutuncu M Tang J Zeid NA Kale N Crusan DJ Atkinson EJ. Onset of progressive phase is an age-dependent clinical milestone in multiple sclerosis. Mult Scler J. (2013) 19:188–98. doi: 10.1177/1352458512451510, PMID: 22736750 PMC4029334

[16] Palmer AL Ousman SS. Astrocytes and aging. Front Aging Neurosci. (2018) 10:337. doi: 10.3389/fnagi.2018.00337 30416441 PMC6212515

[17] Macaron G Larochelle C Arbour N Galmard M Girard JM Prat A. Impact of aging on treatment considerations for multiple sclerosis patients. Front Neurol. (2023) 14:1197212. doi: 10.3389/fneur.2023.1197212, PMID: 37483447 PMC10361071

[18] Lublin FD Reingold SC Cohen JA Cutter GR Sorensen PS Thompson AJ. Defining the clinical course of multiple sclerosis: the 2013 revisions. Neurology. (2014) 83:278–86. doi: 10.1212/WNL.0000000000000560, PMID: 24871874 PMC4117366

[19] Miller DH Chard DT Ciccarelli O. Clinically isolated syndromes. Lancet Neurol. (2012) 11:157–69. doi: 10.1016/S1474-4422(11)70274-5 22265211

[20] Klineova S Lublin FD. Clinical course of multiple sclerosis. Cold Spring Harb Perspect Med. (2018) 8:a028928. doi: 10.1101/cshperspect.a028928, PMID: 29358317 PMC6120692

[21] Lublin FD Baier M Cutter G. Effect of relapses on development of residual deficit in multiple sclerosis. Neurology. (2003) 61:1528–32. doi: 10.1212/01.WNL.0000096175.39831.21 14663037

[22] Rovaris M Confavreux C Furlan R Kappos L Comi G Filippi M. Secondary progressive multiple sclerosis: current knowledge and future challenges. Lancet Neurol. (2006) 5:343–54. doi: 10.1016/S1474-4422(06)70410-0 16545751

[23] Compston A Coles A. Multiple sclerosis. Lancet. (2008) 372:1502–17. doi: 10.1016/S0140-6736(08)61620-7 18970977

[24] Ransohoff RM Hafler DA Lucchinetti CF. Multiple sclerosis—a quiet revolution. Nat Rev Neurol. (2015) 11:134–42. doi: 10.1038/nrneurol.2015.14, PMID: 25686758 PMC4556342

[25] Kremenchutzky M Rice GPA Baskerville J Wingerchuk DM Ebers GC. The natural history of multiple sclerosis: a geographically based study 9: observations on the progressive phase of the disease. Brain. (2006) 129:584–94. doi: 10.1093/brain/awh721 16401620

[26] Kuhlmann T Moccia M Coetzee T Cohen JA Correale J Graves J. Multiple sclerosis progression: time for a new mechanism-driven framework. Lancet Neurol. (2023) 22:78–88. doi: 10.1016/S1474-4422(22)00289-7, PMID: 36410373 PMC10463558

[27] McGinley MP Goldschmidt CH Rae-Grant AD. Diagnosis and treatment of multiple sclerosis: a review. JAMA. (2021) 325:765. doi: 10.1001/jama.2020.26858 33620411

[28] Melamed E Palmer JL Fonken C. Advantages and limitations of experimental autoimmune encephalomyelitis in breaking down the role of the gut microbiome in multiple sclerosis. Front Mol Neurosci. (2022) 15:1019877. doi: 10.3389/fnmol.2022.1019877, PMID: 36407764 PMC9672668

[29] Boziki M Theotokis P Kesidou E Karafoulidou E Konstantinou C Michailidou I. Sex, aging and immunity in multiple sclerosis and experimental autoimmune encephalomyelitis: an intriguing interaction. Front Neurol. (2023) 13:1104552. doi: 10.3389/fneur.2022.1104552, PMID: 36698908 PMC9869255

[30] Constantinescu CS Farooqi N O’Brien K Gran B. Experimental autoimmune encephalomyelitis (EAE) as a model for multiple sclerosis (MS): EAE as model for MS. Br J Pharmacol. (2011) 164:1079–106. doi: 10.1111/j.1476-5381.2011.01302.x, PMID: 21371012 PMC3229753

[31] Pöllinger B Krishnamoorthy G Berer K Lassmann H Bösl MR Dunn R. Spontaneous relapsing-remitting EAE in the SJL/J mouse: MOG-reactive transgenic T cells recruit endogenous MOG-specific B cells. J Exp Med. (2009) 206:1303–16. doi: 10.1084/jem.20090299, PMID: 19487416 PMC2715069

[32] Zhang H Kim Y Ro EJ Ho C Lee D Trapp BD. Hippocampal neurogenesis and neural circuit formation in a Cuprizone-induced multiple sclerosis mouse model. J Neurosci. (2020) 40:447–58. doi: 10.1523/JNEUROSCI.0866-19.2019, PMID: 31719166 PMC6948946

[33] Pike SC Welsh N Linzey M Gilli F. Theiler’s virus-induced demyelinating disease as an infectious model of progressive multiple sclerosis. Front Mol Neurosci. (2022) 15:1019799. doi: 10.3389/fnmol.2022.1019799, PMID: 36311024 PMC9606571

[34] Gerhauser I Hansmann F Ciurkiewicz M Löscher W Beineke A. Facets of Theiler’s murine encephalomyelitis virus-induced diseases: an update. Int J Mol Sci. (2019) 20:448. doi: 10.3390/ijms20020448, PMID: 30669615 PMC6358740

[35] Høglund RA. Multiple sclerosis and the role of immune cells. World J Exp Med. (2014) 4:27–37. doi: 10.5493/wjem.v4.i3.27, PMID: 25254187 PMC4172701

[36] Charo IF Ransohoff RM. The many roles of chemokines and chemokine receptors in inflammation. N Engl J Med. (2006) 354:610–21. doi: 10.1056/NEJMra052723 16467548

[37] Engelhardt B Ransohoff RM. The ins and outs of T-lymphocyte trafficking to the CNS: anatomical sites and molecular mechanisms. Trends Immunol. (2005) 26:485–95. doi: 10.1016/j.it.2005.07.004, PMID: 16039904

[38] Robinson AP Harp CT Noronha A Miller SD. The experimental autoimmune encephalomyelitis (EAE) model of MS: utility for understanding disease pathophysiology and treatment. Handb Clin Neurol. (2014) 122:173–89. doi: 10.1016/B978-0-444-52001-2.00008-X, PMID: 24507518 PMC3981554

[39] Greter M Heppner FL Lemos MP Odermatt BM Goebels N Laufer T. Dendritic cells permit immune invasion of the CNS in an animal model of multiple sclerosis. Nat Med. (2005) 11:328–34. doi: 10.1038/nm1197 15735653

[40] Herz J Zipp F Siffrin V. Neurodegeneration in autoimmune CNS inflammation. Exp Neurol. (2010) 225:9–17. doi: 10.1016/j.expneurol.2009.11.019 19961850

[41] Lassmann H Brück W Lucchinetti CF. The immunopathology of multiple sclerosis: an overview. Brain Pathol. (2007) 17:210–8. doi: 10.1111/j.1750-3639.2007.00064.x, PMID: 17388952 PMC8095582

[42] Wagner CA Roqué PJ Goverman JM. Pathogenic T cell cytokines in multiple sclerosis. J Exp Med. (2020) 217:e20190460. doi: 10.1084/jem.20190460, PMID: 31611252 PMC7037255

[43] Tesmer LA Lundy SK Sarkar S Fox DA. Th17 cells in human disease. Immunol Rev. (2008) 223:87–113. doi: 10.1111/j.1600-065X.2008.00628.x 18613831 PMC3299089

[44] Panitch HS Hirsch RL Schindler J Johnson KP. Treatment of multiple sclerosis with gamma interferon: exacerbations associated with activation of the immune system. Neurology. (1987) 37:1097–7. doi: 10.1212/WNL.37.7.1097 3110648

[45] Ottum PA Arellano G Reyes LI Iruretagoyena M Naves R. Opposing roles of interferon-gamma on cells of the central nervous system in autoimmune Neuroinflammation. Front Immunol. (2015) 6:539. doi: 10.3389/fimmu.2015.00539 26579119 PMC4626643

[46] Fresegna D Bullitta S Musella A Rizzo FR De Vito F Guadalupi L. Re-examining the role of TNF in MS pathogenesis and therapy. Cells. (2020) 9:2290. doi: 10.3390/cells9102290, PMID: 33066433 PMC7602209

[47] Bitsch A. Acute axonal injury in multiple sclerosis: correlation with demyelination and inflammation. Brain. (2000) 123:1174–83. doi: 10.1093/brain/123.6.1174 10825356

[48] Mars LT Bauer J Gross DA Bucciarelli F Firat H Hudrisier D. CD8 T cell responses to myelin oligodendrocyte glycoprotein-derived peptides in humanized HLA-A*0201-transgenic mice. J Immunol. (2007) 179:5090–8. doi: 10.4049/jimmunol.179.8.5090, PMID: 17911594

[49] Selter RC Biberacher V Grummel V Buck D Eienbröker C Oertel WH. Natalizumab treatment decreases serum IgM and IgG levels in multiple sclerosis patients. Mult Scler. (2013) 19:1454–61. doi: 10.1177/1352458513477229, PMID: 23439578

[50] Molnarfi N Schulze-Topphoff U Weber MS Patarroyo JC Prod’homme T Varrin-Doyer M. MHC class II–dependent B cell APC function is required for induction of CNS autoimmunity independent of myelin-specific antibodies. J Exp Med. (2013) 210:2921–37. doi: 10.1084/jem.20130699 24323356 PMC3865476

[51] Sabatino JJ Pröbstel AK Zamvil SS. B cells in autoimmune and neurodegenerative central nervous system diseases. Nat Rev Neurosci. (2019) 20:728–45. doi: 10.1038/s41583-019-0233-2 31712781

[52] van Langelaar J Rijvers L Smolders J van Luijn MM. B and T cells driving multiple sclerosis: identity, Mechanisms and Potential Triggers. Front Immunol. (2020) 11:760. doi: 10.3389/fimmu.2020.00760, PMID: 32457742 PMC7225320

[53] Häusser-Kinzel S Weber MS. The role of B cells and antibodies in multiple sclerosis, Neuromyelitis Optica, and related disorders. Front Immunol. (2019) 10:201. doi: 10.3389/fimmu.2019.00201, PMID: 30800132 PMC6375838

[54] Mimpen M Smolders J Hupperts R Damoiseaux J. Natural killer cells in multiple sclerosis: a review. Immunol Lett. (2020) 222:1–11. doi: 10.1016/j.imlet.2020.02.012 32113900

[55] Baecher-Allan C Kaskow BJ Weiner HL. Multiple sclerosis: mechanisms and immunotherapy. Neuron. (2018) 97:742–68. doi: 10.1016/j.neuron.2018.01.021 29470968

[56] Absinta M Lassmann H Trapp BD. Mechanisms underlying progression in multiple sclerosis. Curr Opin Neurol. (2020) 33:277–85. doi: 10.1097/WCO.0000000000000818, PMID: 32324705 PMC7337978

[57] Lassmann H. Multiple sclerosis pathology. Cold Spring Harb Perspect Med. (2018) 8:a028936. doi: 10.1101/cshperspect.a028936 29358320 PMC5830904

[58] Mahmoud S Gharagozloo M Simard C Gris D. Astrocytes maintain glutamate homeostasis in the CNS by controlling the balance between glutamate uptake and release. Cells. (2019) 8:184. doi: 10.3390/cells8020184, PMID: 30791579 PMC6406900

[59] Haindl MT Köck U Zeitelhofer-Adzemovic M Fazekas F Hochmeister S. The formation of a glial scar does not prohibit remyelination in an animal model of multiple sclerosis. Glia. (2019) 67:467–81. doi: 10.1002/glia.23556, PMID: 30484905 PMC6588096

[60] Kamma E Lasisi W Libner C Ng HS Plemel JR. Central nervous system macrophages in progressive multiple sclerosis: relationship to neurodegeneration and therapeutics. J Neuroinflammation. (2022) 19:45. doi: 10.1186/s12974-022-02408-y, PMID: 35144628 PMC8830034

[61] Ohl K Tenbrock K Kipp M. Oxidative stress in multiple sclerosis: central and peripheral mode of action. Exp Neurol. (2016) 277:58–67. doi: 10.1016/j.expneurol.2015.11.010, PMID: 26626971 PMC7094520

[62] Smith KJ Lassmann H. The role of nitric oxide in multiple sclerosis. Lancet Neurol. (2002) 1:232–41. doi: 10.1016/S1474-4422(02)00102-3 12849456

[63] Campbell GR Ziabreva I Reeve AK Krishnan KJ Reynolds R Howell O. Mitochondrial DNA deletions and neurodegeneration in multiple sclerosis. Ann Neurol. (2011) 69:481–92. doi: 10.1002/ana.22109, PMID: 21446022 PMC3580047

[64] Yamaguchi M Seki T Imayoshi I Tamamaki N Hayashi Y Tatebayashi Y. Neural stem cells and neuro/gliogenesis in the central nervous system: understanding the structural and functional plasticity of the developing, mature, and diseased brain. J Physiol Sci. (2016) 66:197–206. doi: 10.1007/s12576-015-0421-4, PMID: 26578509 PMC4823343

[65] Ming GL Song H. Adult neurogenesis in the mammalian brain: significant answers and significant questions. Neuron. (2011) 70:687–702. doi: 10.1016/j.neuron.2011.05.001, PMID: 21609825 PMC3106107

[66] Martinez-Molina N Kim Y Hockberger P Szele FG. Rostral migratory stream neuroblasts turn and change directions in stereotypic patterns. Cell Adhes Migr. (2011) 5:83–95. doi: 10.4161/cam.5.1.13788, PMID: 21045564 PMC3038103

[67] Hunter M Demarais NJ Faull RLM Grey AC Curtis MA. Layer-specific lipid signatures in the human subventricular zone demonstrated by imaging mass spectrometry. Sci Rep. (2018) 8:2551. doi: 10.1038/s41598-018-20793-4 29416059 PMC5803191

[68] Wang C Liu F Liu YY Zhao CH You Y Wang L. Identification and characterization of neuroblasts in the subventricular zone and rostral migratory stream of the adult human brain. Cell Res. (2011) 21:1534–50. doi: 10.1038/cr.2011.83, PMID: 21577236 PMC3365638

[69] Alvarez-Buylla A Garcia-Verdugo JM. Neurogenesis in adult subventricular zone. J Neurosci. (2002) 22:629–34. doi: 10.1523/JNEUROSCI.22-03-00629.2002, PMID: 11826091 PMC6758521

[70] Toda T Parylak SL Linker SB Gage FH. The role of adult hippocampal neurogenesis in brain health and disease. Mol Psychiatry. (2019) 24:67–87. doi: 10.1038/s41380-018-0036-2 29679070 PMC6195869

[71] Jurkowski MP Bettio L Woo EK Patten A Yau SY Gil-Mohapel J. Beyond the Hippocampus and the SVZ: adult neurogenesis throughout the brain. Front Cell Neurosci. (2020) 14:576444. doi: 10.3389/fncel.2020.576444, PMID: 33132848 PMC7550688

[72] Gonçalves JT Schafer ST Gage FH. Adult neurogenesis in the Hippocampus: from stem cells to behavior. Cell. (2016) 167:897–914. doi: 10.1016/j.cell.2016.10.021 27814520

[73] Rusanescu G. Adult spinal cord neurogenesis: a regulator of nociception. Neurogenesis. (2016) 3:e1256853. doi: 10.1080/23262133.2016.1256853, PMID: 28405586 PMC5384611

[74] Decimo I Bifari F Rodriguez FJ Malpeli G Dolci S Lavarini V. Nestin-and doublecortin-positive cells reside in adult spinal cord meninges and participate in injury-induced parenchymal reaction. Stem Cells. (2011) 29:2062–76. doi: 10.1002/stem.766, PMID: 22038821 PMC3468739

[75] Martens DJ Seaberg RM van der Kooy D. In vivo infusions of exogenous growth factors into the fourth ventricle of the adult mouse brain increase the proliferation of neural progenitors around the fourth ventricle and the central canal of the spinal cord. Eur J Neurosci. (2002) 16:1045–57. doi: 10.1046/j.1460-9568.2002.02181.x, PMID: 12383233

[76] Rodríguez-Barrera R Rivas-González M García-Sánchez J Mojica-Torres D Ibarra A. Neurogenesis after spinal cord injury: state of the art. Cells. (2021) 10:1499. doi: 10.3390/cells10061499, PMID: 34203611 PMC8232196

[77] Iroegbu JD Ijomone OK Femi-Akinlosotu OM Ijomone OM. ERK/MAPK signalling in the developing brain: perturbations and consequences. Neurosci Biobehav Rev. (2021) 131:792–805. doi: 10.1016/j.neubiorev.2021.10.009, PMID: 34634357

[78] Nakatani H Martin E Hassani H Clavairoly A Maire CL Viadieu A. Ascl 1/mash 1 promotes brain Oligodendrogenesis during myelination and Remyelination. J Neurosci. (2013) 33:9752–68. doi: 10.1523/JNEUROSCI.0805-13.2013, PMID: 23739972 PMC3892435

[79] Lopez Juarez A He D Richard LQ. Oligodendrocyte progenitor programming and reprogramming: toward myelin regeneration. Brain Res. (2016) 1638:209–20. doi: 10.1016/j.brainres.2015.10.051, PMID: 26546966 PMC5119932

[80] Kohyama J Kojima T Takatsuka E Yamashita T Namiki J Hsieh J. Epigenetic regulation of neural cell differentiation plasticity in the adult mammalian brain. Proc Natl Acad Sci U S A. (2008) 105:18012–7. doi: 10.1073/pnas.0808417105, PMID: 19004774 PMC2584744

[81] Knight J Hackett C Breton J Mao-Draayer Y. Cross-talk between CD4+ T-cells and neural stem/progenitor cells. J Neurol Sci. (2011) 306:121–8. doi: 10.1016/j.jns.2011.03.030, PMID: 21492879 PMC13544357

[82] Wang T Lee MH Johnson T Allie R Hu L Calabresi PA. Activated T-cells inhibit neurogenesis by releasing granzyme B: rescue by Kv1.3 blockers. J Neurosci. (2010) 30:5020–7. doi: 10.1523/JNEUROSCI.0311-10.2010, PMID: 20371822 PMC2878660

[83] Nikolakopoulou AM Dutta R Chen Z Miller RH Trapp BD. Activated microglia enhance neurogenesis via trypsinogen secretion. Proc Natl Acad Sci USA. (2013) 110:8714–9. doi: 10.1073/pnas.1218856110, PMID: 23650361 PMC3666689

[84] Butovsky O Ziv Y Schwartz A Landa G Talpalar AE Pluchino S. Microglia activated by IL-4 or IFN-γ differentially induce neurogenesis and oligodendrogenesis from adult stem/progenitor cells. Mol Cell Neurosci. (2006) 31:149–60. doi: 10.1016/j.mcn.2005.10.006 16297637

[85] Picard-Riera N Decker L Delarasse C Goude K Nait-Oumesmar B Liblau R. Experimental autoimmune encephalomyelitis mobilizes neural progenitors from the subventricular zone to undergo oligodendrogenesis in adult mice. Proc Natl Acad Sci U S A. (2002) 99:13211–6. doi: 10.1073/pnas.192314199, PMID: 12235363 PMC130612

[86] Tepavčević V Lazarini F Alfaro-Cervello C Kerninon C Yoshikawa K Garcia-Verdugo JM. Inflammation-induced subventricular zone dysfunction leads to olfactory deficits in a targeted mouse model of multiple sclerosis. J Clin Invest. (2011) 121:4722–34. doi: 10.1172/JCI59145, PMID: 22056384 PMC3226002

[87] Mecha M Feliú A Carrillo-Salinas FJ Mestre L Guaza C. Mobilization of progenitors in the subventricular zone to undergo oligodendrogenesis in the Theiler’s virus model of multiple sclerosis: implications for remyelination at lesions sites. Exp Neurol. (2013) 250:348–52. doi: 10.1016/j.expneurol.2013.10.011, PMID: 24148569

[88] Giannakopoulou A Grigoriadis N Bekiari C Lourbopoulos A Dori I Tsingotjidou AS. Acute inflammation alters adult hippocampal neurogenesis in a multiple sclerosis mouse model. J Neurosci Res. (2013) 91:890–900. doi: 10.1002/jnr.23226, PMID: 23606574

[89] Giannakopoulou A Lyras GA Grigoriadis N. Long-term effects of autoimmune CNS inflammation on adult hippocampal neurogenesis. J Neurosci Res. (2017) 95:1446–58. doi: 10.1002/jnr.23982, PMID: 27781303

[90] Huehnchen P Prozorovski T Klaissle P Lesemann A Ingwersen J Wolf SA. Modulation of adult hippocampal neurogenesis during myelin-directed autoimmune neuroinflammation. Glia. (2011) 59:132–42. doi: 10.1002/glia.21082, PMID: 20967885

[91] Schneider R Koop B Schröter F Cline J Ingwersen J Berndt C. Activation of Wnt signaling promotes hippocampal neurogenesis in experimental autoimmune encephalomyelitis. Mol Neurodegener. (2016) 11:53. doi: 10.1186/s13024-016-0117-0, PMID: 27480121 PMC4969720

[92] Danilov AI Covacu R Moe MC Langmoen IA Johansson CB Olsson T. Neurogenesis in the adult spinal cord in an experimental model of multiple sclerosis. Eur J Neurosci. (2006) 23:394–400. doi: 10.1111/j.1460-9568.2005.04563.x 16420447

[93] Covacu R Perez Estrada C Arvidsson L Svensson M Brundin L. Change of fate commitment in adult neural progenitor cells subjected to chronic inflammation. J Neurosci. (2014) 34:11571–82. doi: 10.1523/JNEUROSCI.0231-14.2014, PMID: 25164655 PMC6608416

[94] Arvidsson L Covacu R Estrada CP Sankavaram SR Svensson M Brundin L. Long-distance effects of inflammation on differentiation of adult spinal cord neural stem/progenitor cells. J Neuroimmunol. (2015) 288:47–55. doi: 10.1016/j.jneuroim.2015.09.001, PMID: 26531694

[95] Nait-Oumesmar B Picard-Riera N Kerninon C Decker L Seilhean D Höglinger GU. Activation of the subventricular zone in multiple sclerosis: evidence for early glial progenitors. Proc Natl Acad Sci U S A. (2007) 104:4694–9. doi: 10.1073/pnas.0606835104, PMID: 17360586 PMC3025281

[96] Oreja-Guevara C Gómez-Pinedo U García-López J Sánchez-Sánchez R Valverde-Moyano R Rabano-Gutierrez A. Inhibition of neurogenesis in a case of Marburg variant multiple sclerosis. Mult Scler Relat Disord. (2017) 18:71–6. doi: 10.1016/j.msard.2017.09.024, PMID: 29141824

[97] Chang A Smith MC Yin X Fox RJ Staugaitis SM Trapp BD. Neurogenesis in the chronic lesions of multiple sclerosis. Brain. (2008) 131:2366–75. doi: 10.1093/brain/awn157 18669500 PMC2525445

[98] Zhu K Sun J Kang Z Zou Z Wu X Wang Y. Repurposing of omeprazole for oligodendrocyte differentiation and remyelination. Brain Res. (2019) 1710:33–42. doi: 10.1016/j.brainres.2018.12.037 30590025

[99] Aharoni R Arnon R Eilam R. Neurogenesis and neuroprotection induced by peripheral immunomodulatory treatment of experimental autoimmune encephalomyelitis. J Neurosci. (2005) 25:8217–28. doi: 10.1523/JNEUROSCI.1859-05.2005, PMID: 16148229 PMC6725544

[100] Skihar V Silva C Chojnacki A Döring A Stallcup WB Weiss S. Promoting oligodendrogenesis and myelin repair using the multiple sclerosis medication glatiramer acetate. Proc Natl Acad Sci USA. (2009) 106:17992–7. doi: 10.1073/pnas.0909607106, PMID: 19815532 PMC2758287

[101] Filipi M Jack S. Interferons in the treatment of multiple sclerosis. Int J MS Care. (2020) 22:165–72. doi: 10.7224/1537-2073.2018-063, PMID: 32863784 PMC7446632

[102] Hirsch M Knight J Tobita M Soltys J Panitch H Mao-Draayer Y. The effect of interferon-β on mouse neural progenitor cell survival and differentiation. Biochem Biophys Res Commun. (2009) 388:181–6. doi: 10.1016/j.bbrc.2009.07.073, PMID: 19619508 PMC5504408

[103] Arscott WT Soltys J Knight J Mao-Draayer Y. Interferon β-1b directly modulates human neural stem/progenitor cell fate. Brain Res. (2011) 1413:1–8. doi: 10.1016/j.brainres.2011.07.037, PMID: 21855056

[104] Jin M Li Q Gu Y Wan B Huang J Xu X. Leonurine suppresses neuroinflammation through promoting oligodendrocyte maturation. J Cell Mol Med. (2019) 23:1470–85. doi: 10.1111/jcmm.14053, PMID: 30556290 PMC6349161

[105] Khodanovich MY Pishchelko AO Glazacheva VY Pan ES Krutenkova EP Trusov VB. Plant polyprenols reduce demyelination and recover impaired oligodendrogenesis and neurogenesis in the cuprizone murine model of multiple sclerosis. Phytother Res. (2019) 33:1363–73. doi: 10.1002/ptr.6327, PMID: 30864249 PMC6594192

[106] Mei F Mayoral SR Nobuta H Wang F Desponts C Lorrain DS. Identification of the kappa-opioid receptor as a therapeutic target for oligodendrocyte Remyelination. J Neurosci. (2016) 36:7925–35. doi: 10.1523/JNEUROSCI.1493-16.2016, PMID: 27466337 PMC4961778

[107] Feliú A Bonilla Del Río I Carrillo-Salinas FJ Hernández-Torres G Mestre L Puente N. 2-Arachidonoylglycerol reduces proteoglycans and enhances Remyelination in a progressive model of demyelination. J Neurosci. (2017) 37:8385–98. doi: 10.1523/JNEUROSCI.2900-16.2017, PMID: 28751457 PMC6596867

[108] Hernández-Torres G Cipriano M Hedén E Björklund E Canales Á Zian D. A reversible and selective inhibitor of Monoacylglycerol lipase ameliorates multiple sclerosis. Angew Chem Int Ed. (2014) 53:13765–70. doi: 10.1002/anie.201407807, PMID: 25298214

[109] Syed YA Zhao C Mahad D Möbius W Altmann F Foss F. Antibody-mediated neutralization of myelin-associated Ephrin B3 accelerates CNS remyelination. Acta Neuropathol. (2016) 131:281–98. doi: 10.1007/s00401-015-1521-1 26687980 PMC4713754

[110] Zhang J Zhang ZG Morris D Li Y Roberts C Elias SB. Neurological functional recovery after thymosin beta 4 treatment in mice with experimental auto encephalomyelitis. Neuroscience. (2009) 164:1887–93. doi: 10.1016/j.neuroscience.2009.09.054, PMID: 19782721 PMC2784109

[111] Rafei M Birman E Forner K Galipeau J. Allogeneic mesenchymal stem cells for treatment of experimental autoimmune encephalomyelitis. Mol Ther. (2009) 17:1799–803. doi: 10.1038/mt.2009.157, PMID: 19602999 PMC2835011

[112] Bai L Lennon DP Eaton V Maier K Caplan AI Miller SD. Human bone marrow-derived mesenchymal stem cells induce Th2-polarized immune response and promote endogenous repair in animal models of multiple sclerosis. Glia. (2009) 57:1192–203. doi: 10.1002/glia.20841, PMID: 19191336 PMC2706928

[113] Bai L Lennon DP Caplan AI DeChant A Hecker J Kranso J. Hepatocyte growth factor mediates mesenchymal stem cell–induced recovery in multiple sclerosis models. Nat Neurosci. (2012) 15:862–70. doi: 10.1038/nn.3109, PMID: 22610068 PMC3427471

[114] Barhum Y Gai-Castro S Bahat-Stromza M Barzilay R Melamed E Offen D. Intracerebroventricular transplantation of human mesenchymal stem cells induced to secrete neurotrophic factors attenuates clinical symptoms in a mouse model of multiple sclerosis. J Mol Neurosci. (2010) 41:129–37. doi: 10.1007/s12031-009-9302-8, PMID: 19902385

[115] Constantin G Marconi S Rossi B Angiari S Calderan L Anghileri E. Adipose-derived mesenchymal stem cells ameliorate chronic experimental autoimmune encephalomyelitis. Stem Cells. (2009) 27:2624–35. doi: 10.1002/stem.194 19676124

[116] Razavi S Ghasemi N Mardani M Salehi H. Co-transplantation of human neurotrophic factor secreting cells and adipose-derived stem cells in rat model of multiple sclerosis. Cell J. (2018) 20:46–52. doi: 10.22074/cellj.2018.4777, PMID: 29308618 PMC5759680

[117] Ganji R Razavi S Ghasemi N Mardani M. Improvement of Remyelination in demyelinated Corpus callosum using human adipose-derived stem cells (hADSCs) and Pregnenolone in the Cuprizone rat model of multiple sclerosis. J Mol Neurosci. (2020) 70:1088–99. doi: 10.1007/s12031-020-01515-w, PMID: 32314194

[118] Mardani M Ganji R Ghasemi N Kazemi M Razavi S. Impact of intraventricular human adipose-derived stem cells transplantation with Pregnenolone treatment on Remyelination of Corpus callosum in a rat model of multiple sclerosis. Cell J. (2022) 24:748–56. doi: 10.22074/cellj.2022.8173 PMID: 36527347 PMC9790074

[119] Clark K Zhang S Barthe S Kumar P Pivetti C Kreutzberg N. Placental mesenchymal stem cell-derived extracellular vesicles promote myelin regeneration in an animal model of multiple sclerosis. Cells. (2019) 8:1497. doi: 10.3390/cells8121497, PMID: 31771176 PMC6952942

[120] Brown C McKee C Halassy S Kojan S Feinstein DL Chaudhry GR. Neural stem cells derived from primitive mesenchymal stem cells reversed disease symptoms and promoted neurogenesis in an experimental autoimmune encephalomyelitis mouse model of multiple sclerosis. Stem Cell Res Ther. (2021) 12:499. doi: 10.1186/s13287-021-02563-8, PMID: 34503569 PMC8427882

[121] Shu J He X Li H Liu X Qiu X Zhou T. The beneficial effect of human amnion mesenchymal cells in inhibition of inflammation and induction of neuronal repair in EAE mice. J Immunol Res. (2018) 2018:1–10. doi: 10.1155/2018/5083797, PMID: 30035132 PMC6035808

[122] Lindsay SL Molęda AM Mac Lellan LM Keh SM McElroy DE Linington C. Human olfactory mesenchymal stromal cell transplantation ameliorates experimental autoimmune encephalomyelitis revealing an inhibitory role for IL16 on myelination. Acta Neuropathol Commun. (2022) 10:12. doi: 10.1186/s40478-022-01316-9, PMID: 35093166 PMC8800340

[123] Cecerska-Heryć E Pękała M Serwin N Gliźniewicz M Grygorcewicz B Michalczyk A. The use of stem cells as a potential treatment method for selected neurodegenerative diseases: review. Cell Mol Neurobiol. (2023) 43:2643–73. doi: 10.1007/s10571-023-01344-6, PMID: 37027074 PMC10333383

